# Associations Between Plant-Derived Diet Composition and Fecal Microbiota in Wintering Common Cranes in the Yellow River Estuary

**DOI:** 10.3390/biology15201847

**Published:** 2026-10-15

**Authors:** Miaomiao Yan, Yue Qi, Xiaoxin Liu, Yuanyuan Li, Tianfeng Liu, Ji Feng, Yuchen Li, Yang Zhang

**Affiliations:** 1 State Key Laboratory of Environmental Criteria and Risk Assessment, Chinese Research Academy of Environmental Sciences, Beijing 100012, China; ymm20001113@163.com (M.Y.); lx2920855373@163.com (X.L.); 18134162709@163.com (T.L.); feng.ji@craes.org.cn (J.F.); li.yuchen@craes.org.cn (Y.L.); 2 College of Water Sciences, Beijing Normal University, Beijing 100875, China; lyy20012022@163.com; 3 Laboratory of Ecological Civilization and Social Governance, Chinese Research Academy of Environmental Sciences, Beijing 100012, China; 4 Jiangxi Academy of Forestry, Nanchang 330013, China; yangzhang3217@163.com

**Keywords:** Common Crane, plant-derived diet, fecal microbiota, DNA metabarcoding, wintering ecology, farmland foraging habitat

## Abstract

The gut microbiota is closely linked to nutrient metabolism and host health in birds, but whether variation in plant-derived diet composition is accompanied by corresponding gut microbiota variation during winter remains unclear. In this exploratory study, we analyzed 150 fecal samples from five groups of wintering Common Cranes (*Grus grus*) in the Yellow River Estuary during the 2025/2026 wintering season. Plant DNA metabarcoding and 16S rRNA sequencing were used to characterize the plant-derived diet composition, fecal microbiota, and predicted microbial functions. Both plant-derived diet composition and fecal microbiota structure differed significantly among groups. Groups J1 and J2, sampled concurrently in January, differed markedly in plant-derived diet composition, whereas overall fecal microbiota separation and differences in most predicted functions were relatively limited. Five plant family–bacterial genus associations passed FDR correction without sampling-group adjustment, but none remained significant after adjustment. Overall, plant-derived diet composition and fecal microbiota both varied across the sampled groups, but their patterns of differentiation were not fully concordant. Because sampling time and site were not fully crossed, these patterns cannot be attributed to diet alone. These findings support considering farmland foraging habitats in the management of wintering Common Cranes, with attention to food resource availability throughout winter.

## Simple Summary

Common Cranes use a variety of food resources during winter, and their fecal microbiota may also differ under different sampling conditions. We analyzed 150 fecal samples collected from five groups of Common Cranes in the Yellow River Estuary during the 2025/2026 wintering season. Plant-derived food composition and fecal microbiota differed among the groups, but the patterns of these differences were not fully consistent. Two groups sampled concurrently in January showed marked differences in plant-derived food composition, whereas differences in their overall fecal microbiota and most predicted microbial functions were relatively limited. Five relationships between plant families and bacterial genera were significant before accounting for sampling-group differences, but none remained significant afterward. These findings do not establish direct dietary effects. Animal-derived foods were not assessed. Farmland plants were important components of the plant-derived food detected in fecal samples, suggesting that management of wintering Common Cranes should take into consideration farmland foraging habitats and the continued availability of food resources.

## 1. Introduction

Cranes (Gruidae) are large waterbirds, and wetlands and surrounding farmland provide important habitats and food resources for wintering populations [1,2]. The Common Crane (*Grus grus*) is widely distributed, with wintering populations occurring in several regions of China [3]. For long-distance migratory birds such as Common Cranes, the wintering period is essential for individual survival, and conditions experienced during this period can influence body condition and the timing of subsequent spring migration [4]. The types, abundance, spatial distribution, and availability of food resources at wintering grounds can influence resource tracking, foraging activities, and energy intake in birds, with potential carry-over effects on subsequent life-history stages [5,6]. Therefore, examining food resource use by Common Cranes during the wintering period is important for understanding their ecological adaptation to wintering conditions and their food resource use strategies.

In the Yellow River Estuary, wetlands and surrounding farmland provide diverse habitats and foraging sites for wintering cranes [7]. Crops and their post-harvest residues represent potential plant-derived food resources for wintering cranes, supplementing those available in natural habitats [1,8]. During the wintering period, food resource availability may vary across the landscape and over time, and the plant-derived diet composition of Common Cranes may also vary across wintering stages [9]. Therefore, examining variation among sampling groups in fecal plant DNA profiles together with fecal microbiota variation may help characterize the correspondence between dietary and microbial variation under natural wintering conditions.

In birds, the gut microbiota has been linked to several physiological processes, including nutrient utilization, energy metabolism, immune regulation, and pathogen defense [10,11]. Both its composition and functional potential may vary with host genetic background, diet composition, habitat, seasonal variation, and physiological state [12,13]. Diet composition can contribute substantially to such variation. Differences among food types may also be related to variation in gut microbiota composition and functional potential through differences in nutrient substrates and food-associated microorganisms. In migratory birds wintering at Poyang Lake, diet composition has been reported to be closely related to gut microbiota composition [14], and shifts in the major food sources of Siberian Cranes coincided with changes in gut microbiota diversity, taxonomic composition, and predicted functions [15]. In sympatric Black-necked Cranes and Common Cranes, differences in plant food diversity were not accompanied by significant differences in gut microbiota composition [12]. Meanwhile, gut microbiota composition can differ among crane species [16,17], and both microbiota composition and diversity may vary within the same species across different wintering regions [18,19]. The gut microbiota of cranes also varies across migration and wintering stages [20,21,22], and pronounced seasonal and interannual variation has been observed in other migratory birds [23]. Taken together, this evidence indicates that the gut microbiota composition of migratory birds is dynamic and may be influenced by multiple factors, including host characteristics, diet, and environmental conditions.

These findings suggest that differences in plant-derived diet composition may not be consistently accompanied by corresponding differences in fecal microbiota. Further observations within a single crane species and wintering region are therefore needed to characterize this correspondence under different sampling conditions. In particular, when plant-derived diet composition differs markedly among groups, it remains unclear whether fecal microbiota structure and predicted functions vary to a similar extent, and whether associations between specific plant and bacterial taxa persist after accounting for group differences. The present study addresses these questions using paired dietary and microbial data from wintering Common Cranes in the Yellow River Estuary. Its contribution lies in describing how dietary and microbial variation correspond across sampling groups and evaluating exploratory plant–microbe associations for further investigation. These observations can inform hypotheses about diet–microbiota relationships for testing in future studies.

To address these questions, we conducted a descriptive and exploratory observational study to characterize variation in plant-derived diet composition and fecal microbiota of Common Cranes across five sampling events in the Yellow River Estuary during the 2025/2026 wintering season and to explore potential associations between the two. Fresh fecal samples were collected for five groups, and plant DNA metabarcoding was combined with bacterial 16S rRNA gene amplicon sequencing to characterize plant-derived diet composition, fecal microbiota structure, and predicted microbial functions. Specifically, we compared plant-derived diet composition and fecal microbiota among the five sampling groups; assessed whether the marked difference in plant-derived diet composition of the two groups sampled concurrently in January was accompanied by corresponding differences in fecal microbiota structure and predicted functions; and examined statistical associations between specific plant families and bacterial genera both before and after adjustment for sampling group. These findings could advance our understanding of the relationship between plant-derived diet variation and fecal microbiota variation during wintering and may inform conservation planning aimed at maintaining suitable farmland foraging habitats and the continued availability of plant food resources for Common Cranes throughout the wintering season in the Yellow River Estuary.

## 2. Materials and Methods

### 2.1. Study Area and Sample Collection

**Figure 1:**
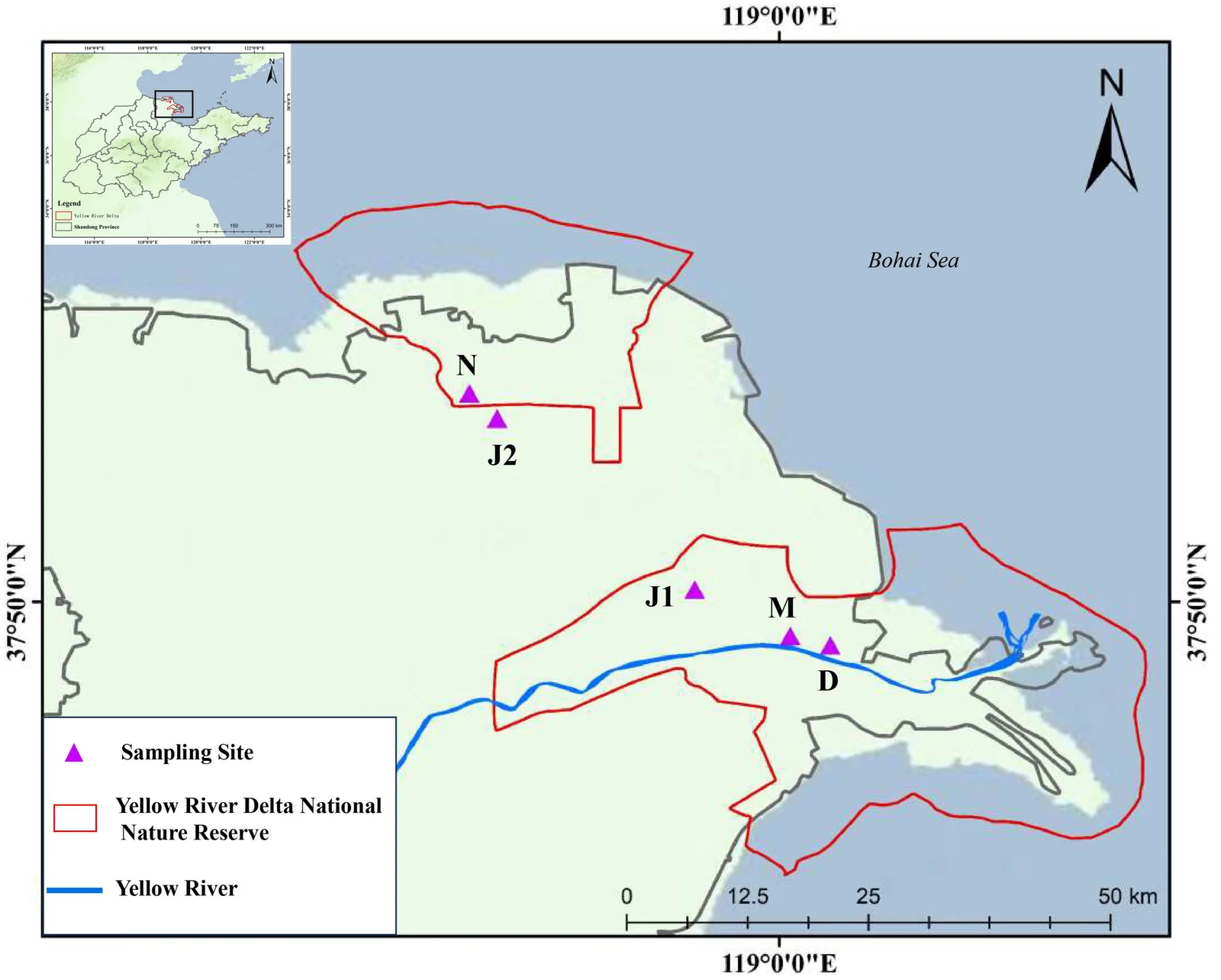
Study area and sampling sites of Common Cranes in the Yellow River Estuary. N, D, J1, J2, and M are the five sampling groups. Terrain basemap source: Tianditu.

The study was conducted in the Yellow River Estuary (Figure 1). Fresh fecal samples were collected non-invasively at farmland foraging sites used by Common Cranes in November and December 2025, and in January and March 2026. Sampling during the wintering period aimed to explore variation in plant-derived diet composition and fecal microbiota. Specific sampling events were arranged according to the presence and foraging activity of crane flocks. Most sampling sites were located near either the former channel of the Yellow River, which ceased to serve as the main channel after the Yellow River shifted course in 1976, or the current channel. In January 2026, samples were collected from two local foraging sites during the same sampling period. Samples were assigned to five sampling groups (N, D, J1, J2, and M) according to sampling date and site. Each group comprised fecal samples collected on a single day at a distinct farmland site, and each site was sampled only once during the study. Thus, “group” refers to fecal samples collected during a single sampling event, rather than to a flock with known individual membership. Pairwise distances among the five sites ranged from approximately 3.4 to 39.3 km (Table S1). Comparisons among groups therefore reflect differences under their respective temporal and spatial conditions, rather than independent temporal, spatial, or crop-specific effects. A total of 208 fecal samples were collected, of which 150 with paired dietary and microbiota data were included in all analyses (Table 1). Of the initially collected samples, 58 (27.9%) were not included in the final paired dataset. Reasons for non-inclusion included non-target species identification, contamination, insufficient fecal material, or dietary amplification failure. For group D, 36 confirmed Common Crane samples meeting the material and quality requirements were selected by simple random sampling without replacement for plant-derived dietary analysis.

Before sampling, crane flocks and their foraging areas were observed using binoculars to estimate flock size, and priority was given to farmland sites used by relatively large flocks. Field personnel entered the foraging area immediately after the cranes had finished foraging and departed. Crane footprints, foraging pits, and the spatial distribution of feces were used to locate sampling areas. To reduce the likelihood of collecting multiple samples from the same individual, only droppings separated by at least 5 m were sampled. Fecal samples were not assigned to individual cranes using host genotyping or other individual-identification methods; therefore, individual identity, sex, age, and kinship were unknown. Repeated sampling of the same individual within or across sampling groups could not be ruled out; therefore, complete independence of samples and groups could not be assured. Freshness was assessed based on the moist and intact appearance of the droppings, and priority was given to those judged to be the freshest. These visual assessments could not establish the exact time since defecation. Samples without visible soil contamination were selected, placed in sterile centrifuge tubes, and transported to the laboratory under chilled conditions. Sampling avoided extreme weather; however, weather conditions, human disturbance, and animal-derived food intake were not quantified or statistically controlled.

### 2.2. DNA Extraction, Polymerase Chain Reaction, and High-Throughput Sequencing

Total genomic DNA was extracted from each fecal sample using an Ezup Column Animal Genomic DNA Purification Kit (Sangon Biotech (Shanghai) Co., Ltd., Shanghai, China) according to the manufacturer’s instructions. DNA integrity was assessed by 1% agarose gel electrophoresis, and DNA concentration and purity were measured using a UV spectrophotometer.

Because Common Cranes and Siberian Cranes foraged in mixed-species flocks in some sampling areas, species identification was performed on fecal samples collected from suspected mixed-species foraging sites to ensure accurate sample origin. We amplified the mitochondrial control region (D-loop) from fecal DNA by polymerase chain reaction (PCR) using the universal primers BZ-For and BZ-Rer for cranes [24]. After agarose gel electrophoresis, we selected suitable PCR products for Sanger sequencing and determined species identity through sequence comparison [12]. Only samples identified as originating from Common Cranes were retained for subsequent analyses of plant-derived diet composition and fecal microbiota.

Given the farmland foraging context of the sampled cranes, dietary profiling focused on plant-derived food components. For plant-derived diet analysis, we used the chloroplast *trnL* sequence as the DNA barcode marker [25], using primers *trnL*-F (CGAAATCGGTAGACGCTACG) and *trnL*-R (CCATTGAGTCTCTGCACCTATC). The *trnL* marker was selected because its conserved primer-binding regions facilitate amplification across diverse plant taxa, making it suitable for fecal plant DNA metabarcoding. Animal-derived dietary components were not assessed, and the resulting plant profiles were not interpreted as representing the complete diet. For fecal microbiota analysis, the V3–V4 hypervariable region of the bacterial 16S rRNA gene was amplified [26] using primers 338F (5′-barcode+ACTCCTACGGGAGGCAGCA-3′) and 806R (5′-GGACTACHVGGGTWTCTAAT-3′). Sequencing libraries were constructed by Personal Biotechnology Co., Ltd. (Shanghai, China), and high-throughput sequencing was performed on an Illumina NovaSeq 6000 platform. PCR negative controls were included during amplification, and samples from different sampling groups were randomly arranged for sequencing.

### 2.3. Bioinformatics and Statistical Analyses

All analyses used the same 150 fecal samples with paired plant-derived dietary and microbiota data (N, *n* = 33; D, *n* = 36; J1, *n* = 24; J2, *n* = 33; M, *n* = 24). Because the five sampling groups (N, D, J1, J2, and M) were jointly defined by sampling time and specific sampling site, and these two factors were not fully crossed in the sampling design, the sampling group was treated as the grouping variable for all subsequent statistical analyses. Differences among the five sampling groups were assessed, without separate statistical inference for sampling time, sampling site, dominant crop, or their interaction. The J1 and J2 groups, both sampled in January, were additionally compared to assess differences between groups sampled within the same month.

Sequencing data from the V3–V4 region of the bacterial 16S rRNA gene were processed using QIIME 2 (version 2024.05) [27]. Raw sequences were demultiplexed, and primer sequences were removed using the cutadapt plugin. The resulting sequences were quality-filtered, denoised, merged, and screened for chimeras using DADA2 [28] to generate an amplicon sequence variant (ASV) feature table and representative sequences. Forward and reverse reads were truncated at 211 bp and 177 bp, respectively, while all other DADA2 parameters were left at their default settings. The resulting amplicon sequence variants (ASVs) were taxonomically assigned using Greengenes Release 13.8 as the reference database, and the taxonomic composition of the fecal microbiota was summarized at the phylum and genus levels. Sequences assigned to chloroplasts or mitochondria and low-abundance features were removed during downstream data filtering. Sequence-processing statistics for individual samples are provided in Supplementary Table S2.

Plant-derived diet composition was analyzed using the chloroplast *trnL* sequencing data. Sequences were subjected to quality control, dereplication, chimera removal, and clustering using VSEARCH (version 2.13.4) [29], and operational taxonomic units (OTUs) were generated at 97% sequence similarity. A regional plant reference list comprising 85 candidate species was compiled based on floristic records of the Yellow River Delta, published studies of Common Crane diets in the study region and other wintering areas, previous field surveys conducted by our research team, and consultations with local nature reserve staff. Representative OTU sequences were compared with reference sequences in the NCBI nucleotide database using BLAST+ version 2.17.0 [30], with the regional plant reference list used to assist taxonomic interpretation. Taxonomic assignments were based on sequence identity and the taxonomic consistency of the best-matching reference sequences. Species-level assignment required ≥99% sequence identity and all equally best-matching references to correspond to a single species. For sequences with >97% but <99% identity, assignment was restricted to the genus level when the best-matching references corresponded to a single species. Within either identity range, equally best-matching references representing multiple species were resolved at their lowest shared taxonomic rank: genus when the species belonged to the same genus, family when they belonged to different genera within the same family. Sequences that could not be reliably identified to the species level were assigned to the lowest reliably supported taxonomic level. After removing non-target taxa and unreliable assignments and filtering out OTUs representing less than 1% of the total reads within each sampling group, a plant taxon abundance matrix was generated for subsequent composition and diversity analyses.

The bacterial ASV and plant *trnL* OTU tables were rarefied to 36,188 and 49,573 reads per sample, respectively. Subsequent filtering of the plant table was not followed by further rarefaction, resulting in different retained read totals among samples. Sample-level read counts before and after filtering are provided in Tables S2 and S3. To assess whether sequencing depth was sufficient for subsequent analyses, Shannon rarefaction curves were generated separately for the *trnL* and 16S rRNA gene datasets. Alpha diversity was evaluated using the Chao1, Observed Species, Shannon, and Simpson indices. Group-specific 95% confidence intervals for median alpha-diversity indices were estimated using 10,000 within-group bootstrap resamples. Overall differences among sampling groups were tested using the Kruskal–Wallis test, followed by Dunn’s post hoc test for pairwise comparisons when the overall test was significant. Beta diversity was assessed using Bray–Curtis dissimilarity [31], and principal coordinates analysis (PCoA) was used to visualize differences in community composition among sampling groups. Homogeneity of multivariate dispersion was assessed using PERMDISP based on distances to group spatial medians with a small-sample bias adjustment. Differences in community composition among sampling groups were tested using permutational multivariate analysis of variance (PERMANOVA) and analysis of similarities (ANOSIM) with 9999 permutations. For ANOSIM, *R* values approaching 1 indicate stronger separation between groups, whereas values near 0 indicate greater overlap. Pairwise comparisons among the five sampling groups were performed, and *p*-values were adjusted using the Benjamini–Hochberg procedure. For the fecal microbiota, linear discriminant analysis effect size (LEfSe) was used to identify taxa that differed among sampling groups, with the linear discriminant analysis (LDA) score threshold set at 4.0 to focus on taxa showing moderately to strongly differentiated effect sizes among sampling groups.

To assess the sensitivity of the results to unequal group sizes, we conducted 1000 repeated subsampling analyses with 24 samples per group. Samples were randomly selected without replacement from groups N, D, and J2, while all samples from J1 and M were retained. For both fecal microbiota and plant-derived diet, alpha-diversity comparisons and Bray–Curtis-based ANOSIM tests were repeated, with Benjamini–Hochberg correction for pairwise comparisons. The results were compared with those from the full dataset to assess the stability of group differences.

Given the limited species-level resolution of the plant *trnL* barcode for some plant taxa, plant abundances were aggregated at the family level for correlation analysis. Bacterial abundances were aggregated at the genus level given the limited species-level resolution of short 16S rRNA amplicons. Using paired dietary and microbiota data from 150 fecal samples, the analyses included all seven plant families detected in the dietary analysis and the ten bacterial genera with the highest overall relative abundances. Plant and bacterial compositions were separately normalized and subjected to centered log-ratio (CLR) transformation after multiplicative zero replacement. Zeros were replaced by 0.65 times each taxon’s smallest positive value, and nonzero components were proportionally rescaled to preserve their ratios and the unit sum. Pearson correlations were calculated for the CLR-transformed data, and sampling-group-adjusted partial correlations were calculated between residuals from taxon-specific linear models with CLR-transformed abundance as the response variable and sampling group as a categorical fixed effect. Significance was assessed using 9999 permutations, unrestricted for unadjusted analyses and restricted within sampling groups for group-adjusted analyses. The Benjamini–Hochberg procedure was jointly applied to all 70 tests within each analysis, with *q* < 0.05 considered significant. Sensitivity to zero replacement was assessed by repeating the group-adjusted analysis with an alternative replacement fraction of 0.50. Sampling-group adjustment and within-group permutations cannot eliminate potential non-independence due to repeated sampling of the same individuals or compensate for the lack of independent sampling-event replication. Accordingly, the analyses focused on describing patterns of association, and all findings were interpreted as exploratory. This analysis reflects statistical associations between plant-derived DNA signals in feces and the relative abundances of bacterial genera; it does not represent actual dietary intake, imply that the detected plant food items were consumed at the fecal collection site, or establish direct causal relationships.

The potential functions of the fecal microbiota were predicted using PICRUSt2 [32] and annotated based on the Kyoto Encyclopedia of Genes and Genomes (KEGG) database, with subsequent analyses conducted at KEGG Level 2. Differences in predicted functional profiles among sampling groups were assessed using the Kruskal–Wallis test, followed by Dunn’s post hoc comparisons, with multiple comparisons corrected using the Benjamini–Hochberg procedure. Data analysis and visualization were performed using R version 4.4.1 and OriginPro 2024b. The principal analytical workflows, software/packages, and procedures are summarized in Supplementary Table S4.

## 3. Results

### 3.1. Sequencing Overview

**Figure 2:**
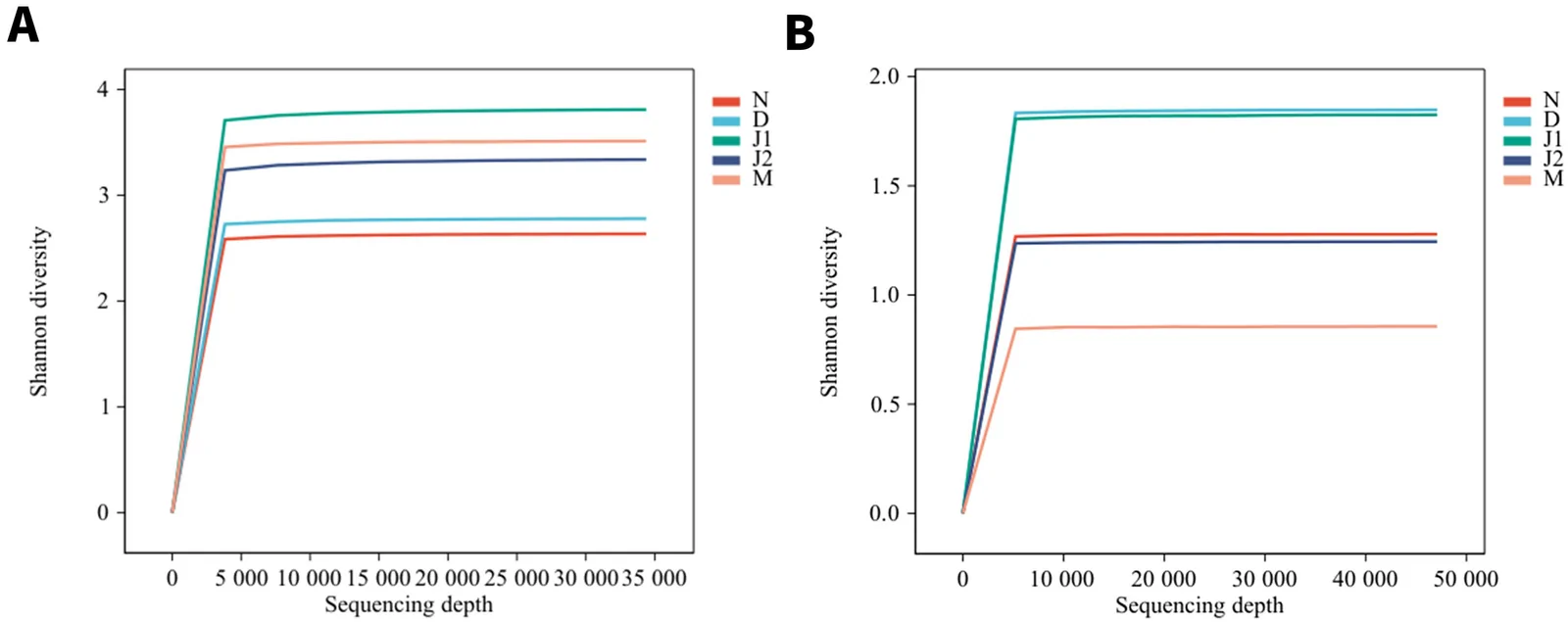
Shannon rarefaction curves for the fecal microbiota (**A**) and plant-derived diet composition (**B**) in the five sampling groups of Common Cranes. Different colors represent different sampling groups.

Sequencing of the bacterial 16S rRNA gene V3–V4 region and plant *trnL* generated 10,580,437 and 14,592,416 valid sequences, respectively. Detailed sample-level sequencing and sequence-processing statistics for the two datasets are provided in Supplementary Tables S2 and S3. Shannon rarefaction curves for both datasets gradually approached a plateau with increasing sequencing depth, indicating that the sequencing depth was adequate for subsequent analyses of the fecal microbiota and plant-derived diet composition of Common Cranes (Figure 2). Sampling information, mean sequencing depths, and principal plant taxa and bacterial phyla for the five sampling groups are summarized in Supplementary Table S5.

### 3.2. Fecal Microbiota Composition and Diversity

**Figure 3:**
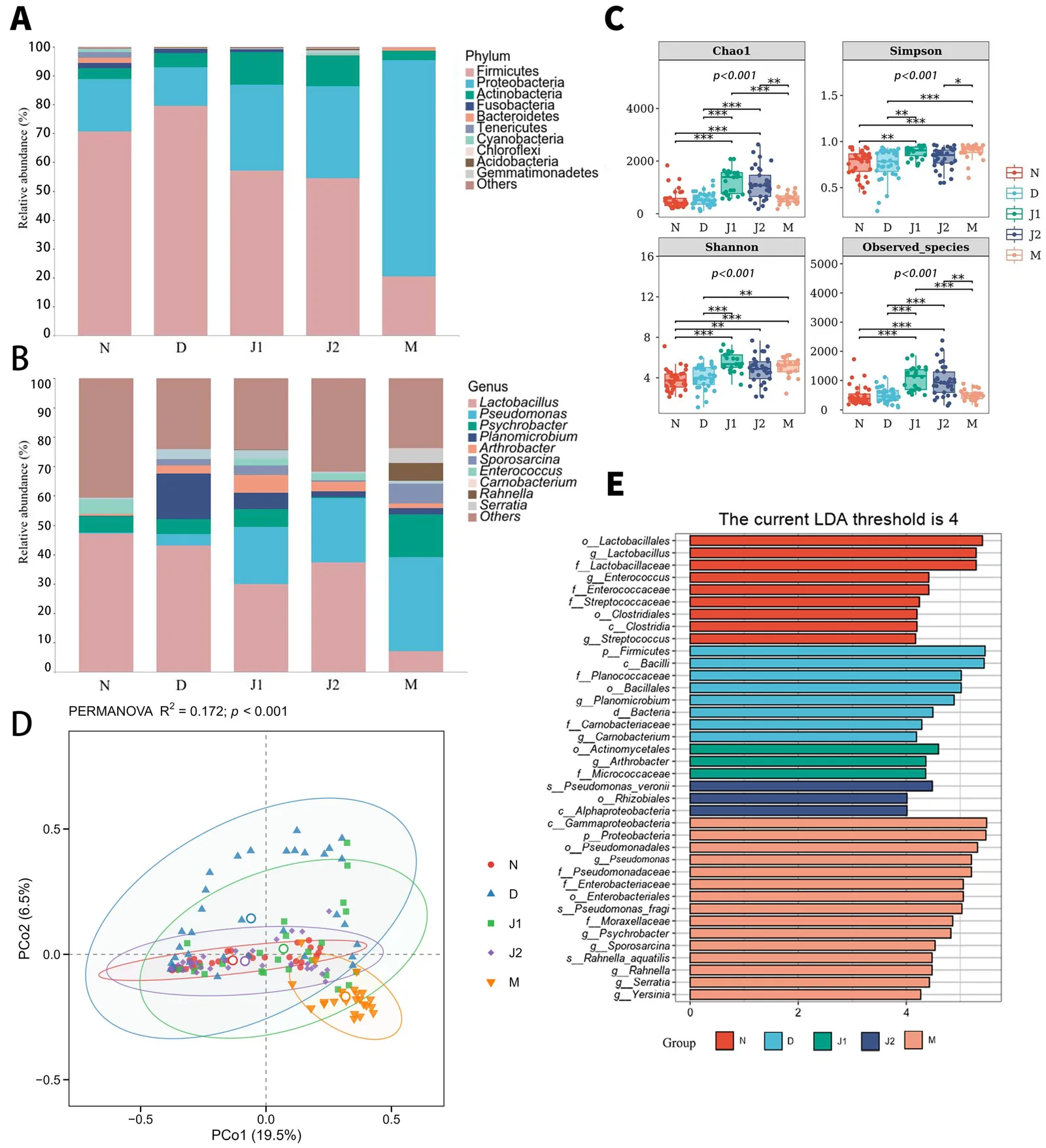
Fecal microbiota composition and diversity among the five sampling groups. (**A**) Phylum-level composition; (**B**) genus-level composition; (**C**) alpha diversity; (**D**) Bray–Curtis-based PCoA; and (**E**) LEfSe analysis. In (**C**), asterisks indicate significant pairwise differences: *p* < 0.05 (*), *p* < 0.01 (**), and *p* < 0.001 (***). In (**D**), points represent individual samples, open circles indicate group centroids, and ellipses represent 95% data ellipses rather than confidence intervals around group centroids.

Across the five sampling groups, the fecal microbiota was dominated by Firmicutes, Proteobacteria, and Actinobacteria, although the relative abundances of these dominant phyla varied among groups (Figure 3A). The following results describe differences among fecal samples collected during five sampling events, defined jointly by sampling time and site; these comparisons are specific to the sampled events and do not isolate temporal, spatial, or dietary effects. Firmicutes showed relatively high abundances in groups N and D, whereas the relative abundance of Proteobacteria was higher in groups J1 and J2 and was particularly high in group M. At the genus level, the dominant genera included *Lactobacillus*, *Pseudomonas*, *Psychrobacter*, *Planomicrobium*, and *Arthrobacter* (Figure 3B). *Lactobacillus* showed relatively high abundance in groups N and D, whereas *Pseudomonas* and *Psychrobacter* were relatively abundant in group M. The prevalence of principal bacterial phyla and genera across the five sampling groups is presented in Supplementary Table S6A.

Alpha diversity analysis revealed significant differences in the Chao1, Observed Species, Shannon, and Simpson indices among the five sampling groups (Kruskal–Wallis test, all *p* < 0.001; Figure 3C). Group-specific alpha-diversity estimates with 95% bootstrap confidence intervals are provided in Table S7. The Chao1 and Observed Species indices were relatively high in groups J1 and J2. In group M, the richness indices were lower than those in groups J1 and J2, whereas the Shannon and Simpson indices were relatively high, indicating that taxon richness and community diversity did not show consistent patterns of variation.

Principal coordinates analysis (PCoA) based on Bray–Curtis dissimilarity showed that PCo1 and PCo2 explained 19.5% and 6.5% of the variation in fecal microbiota structure, respectively, and together accounted for 26.0% of the total variation (Figure 3D). Samples from groups N and J2 showed substantial overlap, whereas group M was more clearly separated from the other sampling groups. PERMDISP did not detect significant differences in multivariate dispersion among the five sampling groups (F_4_,_145_ = 0.341, *p* = 0.8518; Figure S1, left panel). PERMANOVA indicated significant differences in fecal microbiota structure among the sampling groups (pseudo-F_4_,_145_ = 7.545, *R*^2^ = 0.1723, *p* = 0.0001; 9999 permutations), consistent with the ANOSIM result (*R* = 0.3363, *p* = 0.0001). All pairwise PERMANOVA comparisons remained significant after Benjamini–Hochberg correction (adjusted *p* ≤ 0.0004; Table S8). In pairwise ANOSIM comparisons, those involving group M generally yielded relatively high *R* values. Although the difference between groups J1 and J2, which were sampled during the same month, was statistically significant, the overall separation in fecal microbiota structure between the two groups was relatively limited (*R* = 0.2249, adjusted *p* = 0.0003; Table 2).

LEfSe analysis identified characteristic taxa in each of the five sampling groups (LDA score > 4.0; Figure 3E). The taxa characterizing groups N and D mainly included Firmicutes and taxa associated with lactic acid bacteria and *Bacillus*. Taxa characterizing group J1 mainly comprised Actinobacteria-related taxa, whereas those characterizing group J2 mainly included some taxa within Alphaproteobacteria and Rhizobiales. In group M, the characteristic taxa mainly included *Pseudomonas*, *Psychrobacter*, *Rahnella*, *Serratia*, and other taxa within Proteobacteria.

In the balanced subsampling analyses, overall differences among groups remained significant for all four fecal microbiota alpha-diversity indices in all 1000 iterations, although some pairwise comparisons were sensitive to subsampling (Table S9). Overall ANOSIM was also significant in every iteration, with a median R of 0.3638. All pairwise ANOSIM comparisons remained significant after Benjamini–Hochberg correction (Table S10).

### 3.3. Plant-Derived Diet Composition and Diversity

**Figure 4:**
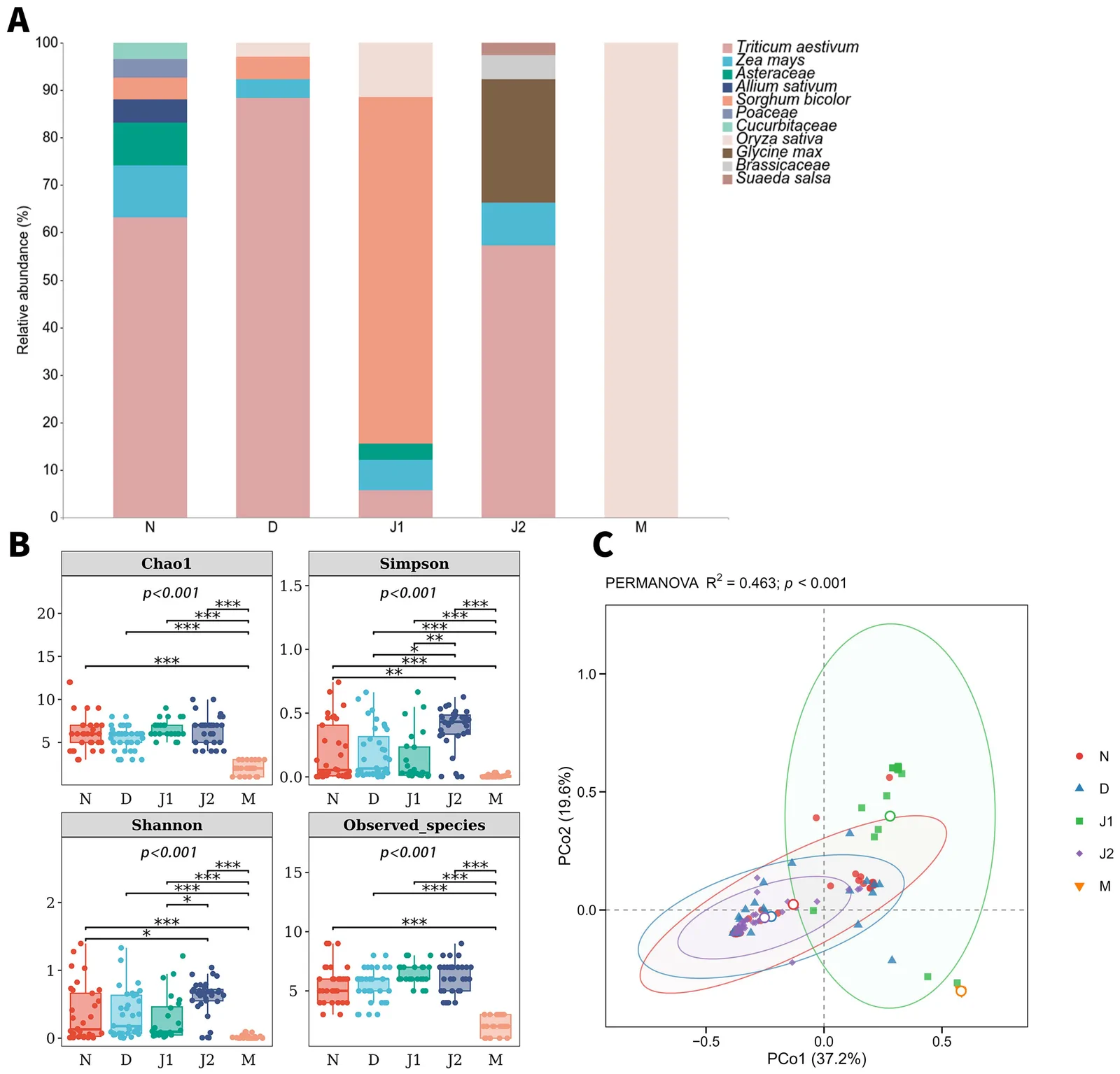
Plant-derived diet composition and diversity among the five sampling groups. (**A**) Relative read abundances of the major plant taxa; (**B**) alpha diversity; and (**C**) Bray–Curtis-based PCoA. In (**B**), asterisks indicate significant pairwise differences: *p* < 0.05 (*), *p* < 0.01 (**), and *p* < 0.001 (***). In (**C**), points represent individual samples, open circles indicate group centroids, and ellipses represent 95% data ellipses rather than confidence intervals around group centroids.

Plant *trnL* sequencing profiles differed markedly among the five sampling groups (Figure 4A). *Triticum aestivum* (common wheat) had the highest relative read abundance in groups N and D. *Sorghum bicolor* (sorghum) had the highest relative read abundance in group J1, with *Oryza sativa* (rice), *Zea mays* (maize), and common wheat also detected. Common wheat and *Glycine max* (soybean) were the predominant taxa in the plant sequence profiles of group J2, whereas rice accounted for the largest proportion of plant reads in group M. The frequency of occurrence of plant taxa across the five sampling groups is presented in Supplementary Table S6B, providing complementary information on the distribution of detected plant taxa among fecal samples.

Alpha diversity analysis revealed significant differences in the Chao1, Observed Species, Shannon, and Simpson indices among the five sampling groups (Kruskal–Wallis test, all *p* < 0.001; Figure 4B). Detailed alpha-diversity estimates are provided in Table S7. Plant-derived dietary richness was relatively high in groups J1 and J2, with group J2 also showing relatively high Shannon and Simpson indices. In contrast, all four diversity indices were relatively low in group M.

Principal coordinates analysis (PCoA) based on Bray–Curtis dissimilarity showed that PCo1 and PCo2 explained 37.2% and 19.6% of the variation in plant-derived diet composition, respectively, and together accounted for 56.8% of the total variation (Figure 4C). The ordination showed overlap among several sampling groups, while group J1 exhibited a relatively broad distribution and group M was relatively separated from the other sampling groups. PERMDISP revealed significant differences in multivariate dispersion among the five sampling groups (F_4_,_145_ = 7.953, *p* = 0.0002; Figure S1, right panel). PERMANOVA also indicated significant differences among sampling groups (pseudo-F_4_,_145_ = 31.224, *R*^2^ = 0.4628, *p* = 0.0001; 9999 permutations), consistent with the ANOSIM result (*R* = 0.5500, *p* = 0.0001). However, the significant differences in multivariate dispersion indicate that the PERMANOVA result may reflect variation in both group composition and within-group dispersion. All pairwise PERMANOVA comparisons remained significant after Benjamini–Hochberg correction (adjusted *p* ≤ 0.0266; Table S11). In pairwise ANOSIM comparisons, groups J1 and J2, which were sampled during the same month, showed a relatively high degree of separation in plant-derived diet composition (*R* = 0.7979, adjusted *p* < 0.001), and comparisons involving group M also generally yielded relatively high *R* values (Table 3). In contrast, the separation in fecal microbiota structure between groups J1 and J2 was relatively limited (*R* = 0.2249, adjusted *p* = 0.0003), and the ANOSIM *R* value was substantially higher for plant-derived diet composition than for fecal microbiota structure. A sensitivity analysis using per-sample relative read abundances yielded a significant overall PERMANOVA result (pseudo-F_4_,_145_ = 42.238, *R*^2^ = 0.5381, *p* = 0.0001), while multivariate dispersion remained heterogeneous (PERMDISP: F_4,145_ = 6.791, *p* = 0.0003).

For the plant-derived diet, overall differences among groups remained significant for all four alpha-diversity indices in all 1000 balanced subsampling iterations, although some pairwise comparisons varied across iterations (Table S9). Overall ANOSIM remained significant throughout, with a median R of 0.6333. All pairwise ANOSIM comparisons remained significant after Benjamini–Hochberg correction except N–D, which was significant in 27.6% of iterations and showed weak separation (median *R* = 0.0236; Table S10).

### 3.4. Correlations Between Plant Families and Dominant Bacterial Genera

Across the 150 matched fecal samples, Pearson correlation analysis of CLR-transformed compositions identified five associations between the seven plant families and ten selected bacterial genera that remained significant after Benjamini–Hochberg correction across all 70 tests (*q* < 0.05; Figure S2A and Table S12). Asteraceae was negatively correlated with *Arthrobacter*, whereas Amaryllidaceae was positively correlated with *Psychrobacter*. Fabaceae was positively correlated with *Arthrobacter* and negatively correlated with *Psychrobacter*, and Poaceae was negatively correlated with *Enterococcus*. These results were obtained without adjustment for sampling group.

**Figure 5:**
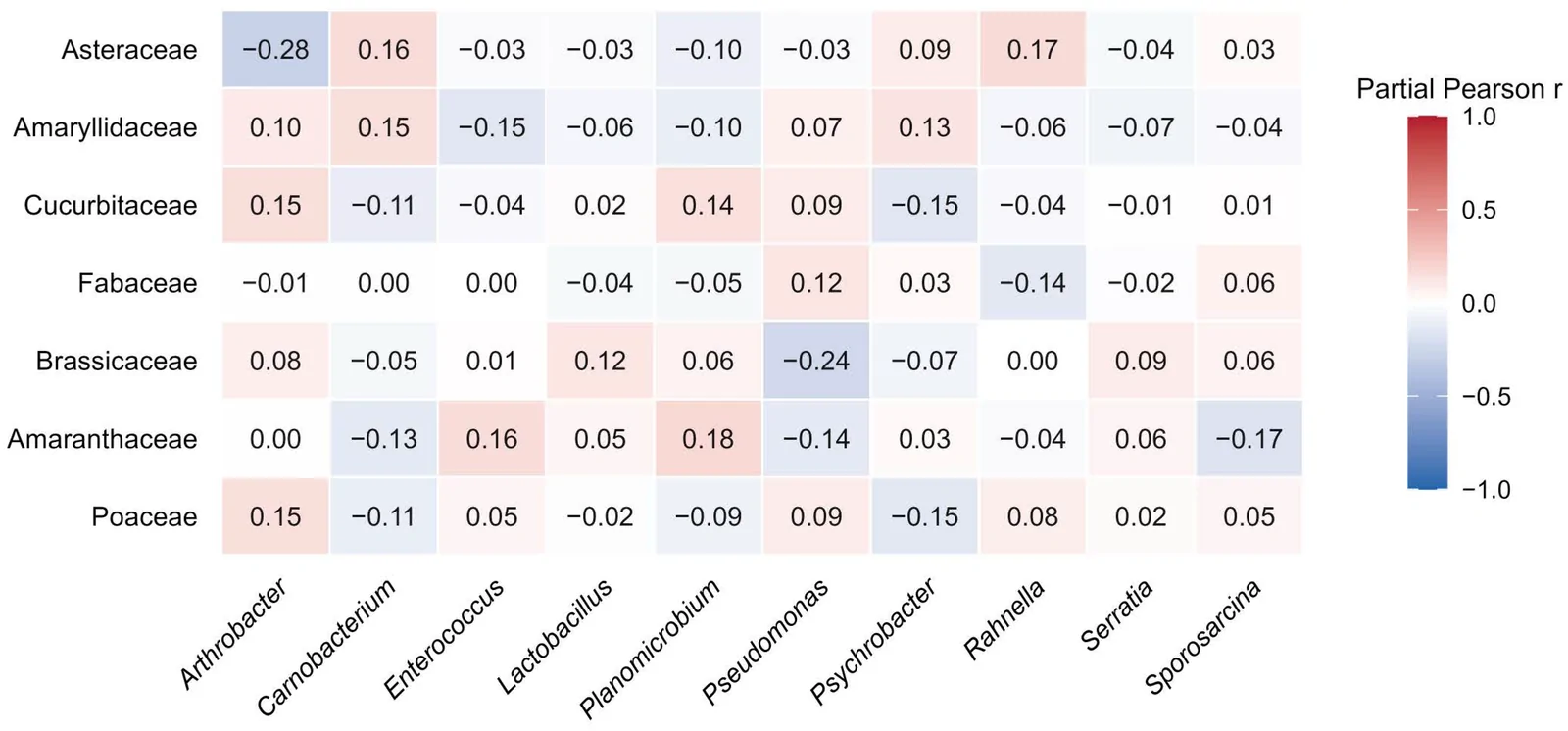
Sampling-group-adjusted associations between plant families and bacterial genera in wintering Common Cranes. Colors and cell values represent partial Pearson correlation coefficients calculated from CLR-transformed compositions after adjustment for sampling group (*n* = 150). Red indicates positive associations, and blue indicates negative associations. No association was significant after Benjamini–Hochberg correction across all 70 tests (all *q* ≥ 0.05).

After adjustment for sampling group, none of the 70 plant-family–bacterial-genus associations remained significant following BH correction (all *q* ≥ 0.05; Figure 5 and Table S12). The smallest adjusted *q* value was observed for Asteraceae–*Arthrobacter* (partial Pearson *r* = −0.279, permutation *p* = 0.0011, *q* = 0.077). Repeating the group-adjusted analysis with an alternative zero-replacement fraction likewise yielded no FDR-significant associations. Thus, the unadjusted associations did not retain statistical significance after sampling-group adjustment and were interpreted as exploratory.

### 3.5. Predicted Functions of the Fecal Microbiota

**Figure 6:**
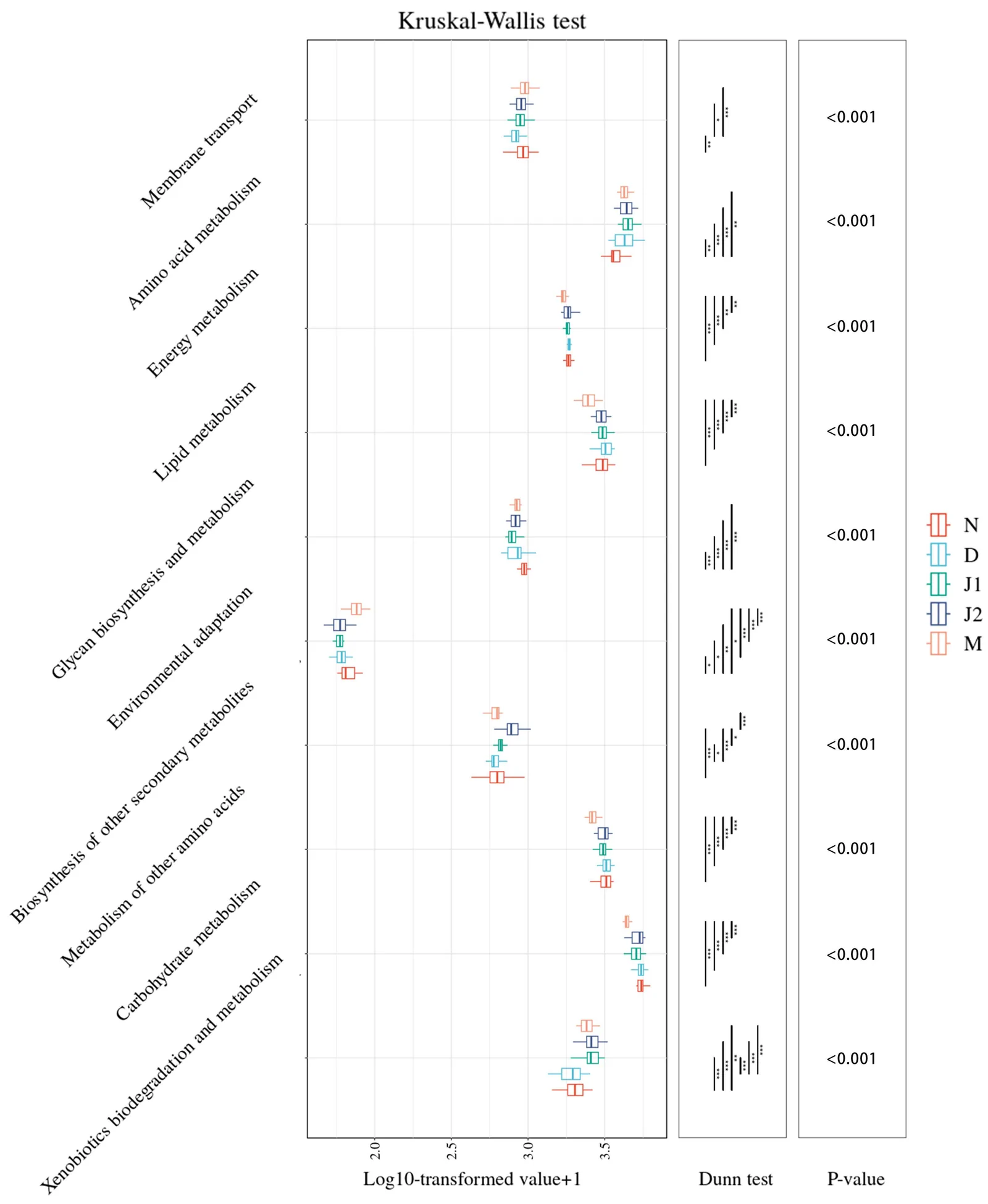
Differences in predicted KEGG Level 2 functional categories of the fecal microbiota among the five sampling groups of Common Cranes. The (**left**) panel shows the predicted relative abundance of each functional category across sampling groups, the (**middle**) panel shows the results of Dunn’s post hoc comparisons, and the (**right**) panel shows the *p*-values from the overall Kruskal–Wallis test. Different colors represent different sampling groups. Asterisks indicate significance levels of pairwise comparisons in Dunn’s post hoc test: *p* < 0.05 (*), *p* < 0.01 (**), and *p* < 0.001 (***).

PICRUSt2 was used to predict the potential functions of the fecal microbiota of Common Cranes, yielding annotations for 36 KEGG Level 2 functional categories. These predictions were inferred from 16S rRNA gene data and do not represent direct measurements of metabolic activity. Based on the Kruskal–Wallis test and after multiple-comparison correction, 10 representative categories showing significant differences and related to nutrient metabolism, energy conversion, metabolism of complex compounds, membrane transport, and environmental adaptation were selected for presentation (Figure 6).

Dunn’s post hoc comparisons revealed significant differences among the sampling groups in PICRUSt2-predicted functional profiles, mainly involving amino acid metabolism, carbohydrate metabolism, lipid metabolism, energy metabolism, environmental adaptation, and xenobiotic biodegradation and metabolism. Group M differed from the other sampling groups in multiple predicted functional categories. In contrast, groups J1 and J2, which were sampled concurrently in January, differed significantly in only one of the 10 functional categories shown: biosynthesis of other secondary metabolites.

## 4. Discussion

The present study documented differences in both plant-derived diet composition and fecal microbiota across five sampling events involving wintering Common Cranes in the Yellow River Estuary, and explored associations between selected plant families and bacterial genera. These findings describe observational patterns rather than direct dietary effects on the microbiota. Groups J1 and J2, which were sampled concurrently in January, differed markedly in plant-derived diet composition, whereas the separation in fecal microbiota structure and differences in most predicted functions between the two groups were relatively limited. These contrasting patterns describe differences between sampling groups but do not, by themselves, establish an association between diet and fecal microbiota. Five plant-family–bacterial-genus associations passed FDR correction in the unadjusted CLR analysis, but none remained significant after sampling-group adjustment. In group M, plant-derived DNA signals were dominated by rice, while its fecal microbiota structure and some predicted functions differed markedly from those of the other sampling groups. This suggests that marked differences in plant-derived diet composition and fecal microbiota may co-occur in some sampling groups, but the observed microbiota differences cannot be directly attributed to plant-derived diet composition.

### 4.1. Composition of and Variation in the Fecal Microbiota in Wintering Common Cranes in the Yellow River Estuary

In the present study, the fecal microbiota across all five sampling groups of Common Cranes was dominated by Firmicutes, Proteobacteria, and Actinobacteria, indicating a broadly consistent composition of dominant phyla across groups. Similar dominant phyla have also been detected in the fecal microbiota of wintering Common Cranes and Siberian Cranes in the Yellow River Estuary [33] and Hooded Cranes wintering at Shengjin Lake [34], suggesting that these phyla may represent important components of the fecal microbiota of wintering crane populations. Some members of Firmicutes possess cellulose-degrading capabilities and may contribute to the digestion and utilization of plant-derived foods [35]. However, the relatively high abundance of Firmicutes across the five sampling groups does not establish their contribution to plant-food digestion, as specific functions cannot be determined from phylum-level relative abundance alone. The bacterial communities identified in fecal samples should be regarded as proxies for distal gut-associated microbiota and may not fully represent microbial communities throughout the gastrointestinal tract. In addition, 16S rRNA amplicon sequencing provides limited taxonomic and functional resolution.

Although the five sampling groups shared similar dominant phyla, the relative abundances of these phyla and the genus-level composition were not entirely consistent. Firmicutes and *Lactobacillus* were relatively abundant in groups N and D, whereas Proteobacteria was clearly dominant in group M, where *Pseudomonas* and *Psychrobacter* also showed relatively high abundances. A similar pattern, in which dominant phyla remain relatively stable while variation occurs at lower taxonomic levels, has also been reported in Hooded Cranes across different migration and wintering stages [36] and in Black-Necked Cranes from different wintering populations [37]. In addition, some *Lactobacillus* strains have been reported to exert probiotic effects, including improvements in intestinal barrier function and immune responses in birds [38]. Therefore, the relatively high abundance of *Lactobacillus* in groups N and D may reflect microbial characteristics associated with the maintenance of gut homeostasis, although the health status of Common Cranes cannot be inferred from genus-level relative abundance alone.

Alpha diversity and community structure analyses further showed that the five sampling groups differed not only in the relative abundances of dominant taxa, but also in fecal microbiota richness and overall community structure. Fecal microbiota richness was relatively high in groups J1 and J2, whereas group M had lower Chao1 and Observed Species indices than these two groups but relatively higher Shannon and Simpson indices. This suggests that although fewer taxa were detected in group M, the distribution of relative abundances among taxa remained relatively even. This pattern may be associated with multiple factors, including plant-derived diet composition, habitat conditions, sampling time, and exposure to environmental microbial sources, rather than any single factor. Although sequencing depth was standardized before diversity analyses, potential sequencing-related effects cannot be completely excluded. PERMANOVA showed that sampling group explained 17.23% of the variation in fecal microbiota structure, indicating significant community differences among sampling groups, while a substantial proportion of the variation may be associated with host individual differences, environmental conditions, and other factors not included in the present study.

Among the five sampling groups, group M showed particularly distinct fecal microbiota characteristics: a clear dominance of Proteobacteria, a relatively low microbiota richness, and clear separation from the other sampling groups in the PCoA ordination space. Samples in group M were collected from a rice-field foraging site in March, and their plant-derived DNA signals were highly concentrated on rice, indicating that marked differences in plant-derived diet composition and fecal microbiota structure co-occurred under this sampling condition. However, this dietary contrast coincided with differences in sampling time and habitat, preventing a separate assessment of dietary and environmental contributions. Variation in avian fecal microbiota has also been associated with seasonal and environmental conditions [39,40]. These findings provide a basis for considering seasonal conditions and foraging habitat alongside diet when interpreting the distinct microbiota of group M. Because temperature, environmental microbial exposure, and host physiological state were not directly assessed, their relative contributions remain unresolved, and the observed microbiota differences cannot be attributed specifically to rice consumption.

Groups J1 and J2, which were sampled concurrently in January, provided additional information comparing fecal microbiota characteristics between groups sampled within the same month. Although the difference in fecal microbiota structure between the two groups was statistically significant, the ANOSIM *R* value was relatively low, indicating limited overall separation. The two groups also showed similar compositions of the dominant phyla, with differences mainly occurring in some bacterial genera and LEfSe-identified taxa. These results indicate that the two sampling groups differed in some aspects of fecal microbiota composition. However, they did not exhibit strong overall community differentiation. Previous studies have likewise reported varying degrees of fecal microbiota differences among Hooded Cranes from different sampling sites and wintering stages [20], Black-necked Cranes from different wintering regions in Yunnan [37], and cranes in different foraging environments in the Yellow River Estuary [7], indicating that spatial and environmental context can be associated with variation in crane fecal microbiota. However, the present comparison between J1 and J2 does not allow the effects of specific spatial or environmental factors to be isolated. Groups J1 and J2 represent only two sampling locations that were sampled within the same month (January); therefore, these results should not be generalized as evidence of widespread spatial differences throughout the Yellow River Estuary.

LEfSe analysis further showed that taxa differing among the five sampling groups were mainly distributed at lower taxonomic levels, such as the family and genus levels, consistent with the finding that the dominant phyla were broadly similar, whereas genus-level composition differed among groups. Both host filtering and environmental microbial input have been reported to contribute to variation in avian fecal microbiota [41]. Host filtering and local environmental conditions may therefore jointly contribute to variation in the fecal microbiota of Common Cranes, allowing some commonality in the dominant phyla while contributing to differences at lower taxonomic levels and in overall community structure.

Overall, the fecal microbiota across the five sampling groups of Common Cranes showed both compositional similarity and marked intergroup variation. However, the study was restricted to a single wintering season and five farmland sampling sites in the Yellow River Estuary, which may limit the generalizability of the findings to other wintering seasons or geographic regions. Each sampling group represented a single sampling event at a distinct site, and sampling time and site were not fully crossed. The observed group differences therefore cannot be independently attributed to temporal, spatial, or crop-specific effects. Although droppings were collected at least 5 m apart, individual identity was not established, and repeated sampling of the same bird could not be excluded. Sex and age class may also influence plant-derived diet composition and fecal microbiota, but their potential effects could not be evaluated because these individual characteristics were unknown. Moreover, the unrestricted sample-level permutation tests did not account for potential dependence among fecal samples within the same sampling event, which may have affected the resulting *p*-values. The 150 fecal samples therefore should not be interpreted as 150 independent replicates of sampling sites or events. These limitations should be considered when interpreting the differences among the sampled groups. Unequal group sizes may also have influenced the statistical comparisons. Balanced subsampling supported the stability of the overall group differences in both fecal microbiota and plant-derived diet, but some pairwise alpha-diversity comparisons and the N–D dietary ANOSIM comparison were sensitive to subsampling (Tables S9 and S10). These weaker pairwise findings therefore require caution. Reduced significance after subsampling may reflect the smaller sample sizes as well as changes in sample composition and cannot be attributed to the original imbalance alone.

### 4.2. Composition of and Variation in Plant-Derived Diet in Wintering Common Cranes in the Yellow River Estuary

In the present study, fecal plant DNA profiles differed markedly among the five sampling groups, with common wheat, sorghum, soybean, and rice representing prominent plant taxa. Common wheat had the highest relative read abundance in groups N and D, sorghum was the most abundant in group J1, common wheat and soybean predominated in group J2, and rice predominated in group M. The dietary differences may partly reflect variation in crop composition and associated food resources among the farmland foraging areas used by the cranes. However, because Common Cranes can move among foraging areas, the plant food items detected in fecal samples may not necessarily have been consumed at the corresponding collection sites.

Groups J1 and J2, which were sampled concurrently in January, differed markedly in plant-derived diet composition. Plant read profiles in group J1 were dominated by sorghum, whereas those in group J2 were dominated by common wheat and soybean. This indicates that substantial heterogeneity in plant-derived diet composition can occur among groups sampled within the same month. Differences in the surrounding agricultural landscapes and crop-resource composition of the two sampling areas may have contributed to this pattern.

Previous studies on cranes have also shown that plant food sources may vary with resource conditions. When aquatic plant resources were insufficient at Caohai, Black-necked Cranes and Common Cranes increased their use of crops such as potatoes [8]. The major plant food sources consumed by Hooded Cranes at Shengjin Lake also varied between different wintering stages [42]. At Poyang Lake, declines in submerged plant resources were accompanied by changes in diet composition and dietary breadth in several crane species, together with increased use of farmland resources [1]. These findings indicate that cranes can adjust their use of different food resources when the types or availability of specific food resources change. Farmland crops and their post-harvest residues can serve as important supplementary food resources for cranes during the wintering period [43]. In the present study, common wheat, sorghum, soybean, and rice were prominent taxa in the fecal plant DNA profiles of Common Cranes, supporting the importance of farmland plant resources to wintering Common Cranes in the Yellow River Estuary. Some foraging sites were located near the boundaries of protected areas or outside them. These observations suggest that conservation planning for wintering Common Cranes should consider not only habitats within protected-area boundaries, but also farmland foraging sites near or outside these boundaries that are actually used by the cranes. Previous studies have shown that residual food resources in farmland may decline as the wintering period progresses, while agricultural activities such as plowing and irrigation may also alter food availability [2,44]. Accordingly, conservation should include attention to the continued availability of food resources and the overall condition of farmland foraging habitats throughout the wintering period, rather than on management of any single crop. More broadly, habitat use by wintering Common Cranes may also be jointly influenced by food availability and accessibility, human disturbance, roost-site security, water conditions, and landscape connectivity; therefore, these broader habitat and landscape factors should also be considered in conservation planning.

Analysis of plant-derived diet diversity showed that groups J1 and J2 had relatively high plant taxon richness, whereas group M had lower richness and diversity, with plant-derived DNA signals dominated by rice. Samples in group M were collected from a rice-field foraging site in March, indicating that the plant-derived diet composition detected in this sampling group was relatively concentrated. However, this result reflects only the plant-derived component of the diet and does not indicate that the complete diet of Common Cranes in group M was similarly restricted. Common Cranes are omnivorous and may consume animal-derived foods in addition to plant-derived foods [45]. Animal-derived foods, including earthworms and insect larvae, have also been documented in the winter diet of Black-necked Cranes [46]. Unmeasured animal-derived foods, including invertebrates, could therefore contribute to variation in fecal microbiota composition and complicate the interpretation of predicted metabolic functions. The nutritional and microbial relevance of animal-derived foods cannot be inferred from plant DNA profiles. This limitation applies to all five sampling groups. The plant *trnL* barcode used in the present study detects only plant-derived DNA. Previous studies using both plant and animal DNA barcodes have detected both types of food sources in fecal samples of wintering Red-Crowned Cranes [47]. Future studies could combine DNA-based detection of animal-derived foods with surveys of food resources at foraging sites to provide a more comprehensive assessment of diet composition and resource use by Common Cranes during the wintering period.

Relative read abundance describes the proportion of sequence reads assigned to a plant taxon and should not be directly equated with its contribution to consumed biomass or nutrient intake. Digestive processes, differences in DNA detectability, amplification bias, and reference-database coverage may affect these proportions [48,49]. Detected plant DNA may originate from consumed seeds, crop residues, or other plant tissues, but incidental ingestion of plant debris and contamination by environmental plant DNA after defecation cannot be excluded. The *trnL* data alone cannot distinguish these sources or identify the plant parts consumed. Furthermore, the limited taxonomic resolution of the *trnL* marker may hinder the discrimination of closely related plant species, particularly certain cultivated crops and their wild relatives.

### 4.3. Association Between Plant-Derived Diet Composition and the Fecal Microbiota in Wintering Common Cranes in the Yellow River Estuary

Plant-derived diet composition and fecal microbiota structure did not show fully consistent patterns of differentiation among the five sampling groups. Group M differed relatively strongly from the other groups in both datasets, whereas J1 and J2 showed marked dietary separation but comparatively limited microbiota separation. Thus, the pronounced dietary differences between some groups were not accompanied by comparably pronounced microbiota differences, suggesting that plant-derived dietary profiles alone may be insufficient to account for the observed microbial variation. This observed contrast does not demonstrate that the microbiota were buffered against effects from dietary changes. Previous avian studies have reported concurrent variation in diet and microbiota [15,50] while recognizing the relevance of host characteristics and environmental conditions [12,51]. Previous studies have considered locality, sampling date, and age when analyzing associations between diet and microbiota [52], and have examined associations of fecal bacterial composition with host traits and habitat characteristics [53]. In the present study, inter-individual variation, host filtering, environmental microbial exposure, and unmeasured animal-derived foods remain possible explanations for the incomplete correspondence. Sequencing biases and limited taxonomic and functional resolution may have also affected the observed patterns. These possibilities cannot be distinguished by the present data and require further investigation through controlled experiments or longitudinal sampling with concurrent environmental measurements.

At the taxonomic level, five plant-family–bacterial-genus associations passed BH-FDR correction in the unadjusted CLR analysis, but none remained significant after adjustment for sampling group. The loss of statistical significance after adjustment does not establish that these associations were spurious or that plant-derived diet composition and fecal microbiota were unrelated because group adjustment also removes plant-derived dietary variation that coincides with sampling context. Rather, the present data provide limited evidence for associations among the selected plant families and bacterial genera beyond sampling-group differences. The unadjusted findings should therefore be regarded as exploratory and require confirmation in sampling designs that better separate dietary variation from temporal and spatial conditions.

Differences in nutrient substrates provide a possible biological basis for associations between plant-derived dietary variation and fecal microbiota. Plants differ in their carbohydrate, protein, fiber, and secondary metabolite composition, which may alter the nutrient substrates entering the digestive tract and thereby be associated with changes in the relative abundances of certain bacterial taxa. Differences in plant fiber structure may favor bacteria with different carbohydrate-degrading capabilities, as demonstrated in vitro for sorghum arabinoxylans [54]. Some plant secondary metabolites, such as flavonoids, may inhibit susceptible bacteria while favoring those capable of metabolizing these compounds [55]. In addition, microorganisms associated with foods and foraging environments may also contribute to variation in fecal microbiota. However, the present study did not directly measure the nutritional composition of foods, environmental microorganisms, or microbial metabolic activity. These mechanisms therefore remain plausible explanations to be tested, rather than mechanisms demonstrated by the present study. The relative abundances of plant DNA sequences may also be affected by amplification bias and the completeness of reference databases. Therefore, they cannot directly represent actual food intake [48,49]. Furthermore, the CLR-based associations describe relative variation within the selected plant families and bacterial genera, not relationships between their absolute abundances. These measurement and analytical limitations, together with the lack of FDR-significant group-adjusted associations, preclude attributing changes in specific bacterial genera to particular plant foods.

From a conservation and management perspective, the observed differences in plant-derived diet composition and fecal microbiota, together with the lack of FDR-significant associations after sampling-group adjustment, do not provide a basis for linking particular plant resources to specific fecal microbial profiles. The detection of agricultural food plants, together with observations of farmland foraging, supports including farmland patches used by cranes within and outside protected areas in wintering habitat management. Monitoring food resources in these patches throughout winter could help identify periods of potential shortage and inform habitat management. Future research should prioritize repeated sampling of multiple sites at comparable times throughout the wintering period, combined with individual identification or tracking, to better distinguish temporal and spatial variation and account for repeated sampling of individuals. Concurrent surveys of food resources and paired analyses of plant-derived and animal-derived diet components and fecal microbiota would help assess whether diet–microbiota associations recur across sites and sampling periods.

### 4.4. Variation in Predicted Functional Profiles of the Fecal Microbiota in Wintering Common Cranes in the Yellow River Estuary

PICRUSt2 predictions showed that the fecal microbiota of Common Cranes differed among the five sampling groups in multiple KEGG Level 2 functional categories. However, the direction of change varied among functional categories. This indicates that differences in the fecal microbiota among sampling groups were reflected not only in taxonomic composition but also in predicted functional profiles. However, these changes did not involve consistent increases or decreases across all functional categories and were instead mainly confined to specific functional categories. Together with the pattern of broadly similar dominant phyla but differences at lower taxonomic levels among the five sampling groups, these results suggest that variation in taxonomic composition may occur concurrently with differences in predicted microbial functions.

Among the predicted functional categories presented in this study, several between-group differences involved categories related to nutrient metabolism, including amino acid, carbohydrate, lipid, and energy metabolism. Given the roles of these functions in dietary nutrient breakdown and utilization, the observed differences may indicate variation in the predicted metabolic potential of the fecal microbiota among sampling groups. A study of sympatrically wintering Black-Necked Cranes and Common Cranes at Caohai also found that differences in plant-derived diet composition were accompanied by differences in some predicted functions related to amino acid and lipid metabolism [8]. These findings suggest that differences in food resource use and variation in the potential metabolic functions of the fecal microbiota may occur concurrently. However, they do not provide evidence of a direct causal relationship between the two. Because the present study did not directly measure microbial functional gene expression or metabolites, these results should still be interpreted as functional predictions based on 16S rRNA data.

The magnitude of differences in predicted functions varied among sampling groups. Although groups J1 and J2, sampled concurrently in January, differed markedly in plant-derived diet composition, most predicted functional categories were similar between the two groups, with a significant difference detected only in the biosynthesis of other secondary metabolites among the selected 10 representative categories shown. This pattern was in line with the relatively limited overall separation in fecal microbiota structure between the two groups. It further indicates that marked differences in plant-derived diet composition are not necessarily accompanied by widespread differences in predicted microbial functions. A study of Black-Necked Cranes also found that differences in predicted fecal microbiota functions between local habitats were smaller than those between different wintering areas [56]. This suggests that the magnitude of variation in predicted microbial functions may be associated with spatial scale, environmental context, and other factors.

Group M showed relatively pronounced differences in multiple predicted functional categories, indicating that distinct predicted functional profiles may occur under different sampling conditions. Previous studies on cranes have shown that extreme environmental conditions or changes in life-history stage can be accompanied by changes in fecal microbiota composition and predicted functions related to metabolism [13,57], suggesting that the potential functions of the fecal microbiota may vary with environmental conditions and life-history stage. Overall, variation in predicted microbial functions did not show a simple correspondence with plant-derived diet composition and may also be associated with host characteristics and environmental context. However, PICRUSt2 provides bioinformatic predictions of potential microbial functions based on 16S rRNA profiles rather than direct measurements of microbial gene content or expression, enzyme activity, metabolites, or metabolic flux. Therefore, these results should not be interpreted as evidence of actual microbial metabolic activity or the physiological or health status of Common Cranes. Confirmation of these inferred functions will require complementary approaches such as shotgun metagenomics, metatranscriptomics, metabolomics, enzyme assays, or targeted microbial culturing.

## 5. Conclusions

In this exploratory study, we observed differences in plant-derived diet composition and fecal microbiota among sampling groups of wintering Common Cranes in the Yellow River Estuary during the 2025/2026 wintering season. However, the patterns of differentiation among groups were not fully consistent. Groups J1 and J2, sampled concurrently in January, differed markedly in plant-derived diet composition, whereas overall separation in fecal microbiota structure and differences in most predicted functions were relatively limited. Five plant-family–bacterial-genus associations passed FDR correction in the unadjusted CLR analysis, but none remained significant after adjustment for sampling group. The findings do not establish that particular plant foods caused the observed microbiota differences or that the microbiota were resistant to dietary change. In group M, rice-dominated plant DNA profiles coincided with pronounced differences in fecal microbiota structure and predicted functions, but these microbiota differences cannot be attributed to a single dietary factor. Because sampling time and site were not fully crossed, differences among the five sampling groups cannot be interpreted as independent temporal, spatial, or dietary effects. These conclusions concern only the plant-derived component of the diet, and the contribution of animal-derived foods to the observed microbiota patterns remains unresolved.

Common wheat, sorghum, soybean, and rice were prominent taxa in the fecal plant DNA profiles. Together with observations of farmland foraging, these detections support the relevance of farmland plant resources to wintering Common Cranes, although their quantitative contributions to food intake could not be established. These findings support considering farmland foraging patches used by Common Cranes, including those near or outside protected-area boundaries, in wintering habitat management, with attention to food-resource availability throughout winter.

Future studies combining spatially and temporally replicated sampling with more comprehensive dietary assessments, including animal-derived foods, could help evaluate the consistency of diet–microbiota associations across sites and periods. Metagenomic and metabolomic approaches could also be incorporated [58,59] to test the predicted functional differences among sampling groups.

## Figures and Tables

**Table 1:** Basic information on the five sampling groups.

Group	Sampling Month	Locality	Habitat	Main Crops	Collected n	Analyzed n
N	November 2025	Former Yellow River channel	Farmland	Maize; common wheat	44	33
D	December 2025	Current Yellow River channel	Farmland	Common wheat	79	36
J1	January 2026	Current Yellow River channel	Farmland	Sorghum	25	24
J2	January 2026	Former Yellow River channel	Farmland	Common wheat	36	33
M	March 2026	Current Yellow River channel	Farmland	Rice	24	24

Note: Named crops include standing crops, post-harvest residues, and recently sown crops recorded during field observations.

**Table 2:** Overall and pairwise ANOSIM results for fecal microbiota structure among the five sampling groups.

Group 1	Group 2	*R*	*p*	*p*_adj_
All	–	0.3363	0.0001	-
N	D	0.1689	0.0001	0.0001
N	J1	0.2800	0.0001	0.0001
N	J2	0.1468	0.0001	0.0001
N	M	0.7089	0.0001	0.0001
D	J1	0.1715	0.0014	0.0014
D	J2	0.1973	0.0001	0.0001
D	M	0.6205	0.0001	0.0001
J1	J2	0.2249	0.0003	0.0003
J1	M	0.4702	0.0001	0.0001
J2	M	0.7646	0.0001	0.0001

Note: Bray–Curtis dissimilarities were calculated from ASV abundances. Pairwise *p*-values were adjusted using the Benjamini–Hochberg method.

**Table 3:** Overall and pairwise ANOSIM results for plant-derived diet composition among the five sampling groups.

Group 1	Group 2	*R*	*p*	*p*_adj_
All	–	0.5500	0.0001	-
N	D	0.0435	0.0194	0.0194
N	J1	0.4783	0.0001	0.0001
N	J2	0.0931	0.0004	0.0005
N	M	0.9129	0.0001	0.0001
D	J1	0.6419	0.0001	0.0001
D	J2	0.0954	0.0008	0.0009
D	M	0.9206	0.0001	0.0001
J1	J2	0.7979	0.0001	0.0001
J1	M	0.8424	0.0001	0.0001
J2	M	0.9873	0.0001	0.0001

Note: Bray–Curtis dissimilarities were calculated from plant OTU abundances. Pairwise *p*-values were adjusted using the Benjamini–Hochberg method.

## Data Availability

The raw sequencing data for both bacterial 16S rRNA gene and plant *trnL* amplicons generated in this study have been deposited in the NCBI Sequence Read Archive (SRA) under BioProject accession number PRJNA1520840. The data will be made publicly available upon publication of the article. The bacterial ASV and plant *trnL* OTU abundance tables with taxonomic assignments, together with non-sensitive sample metadata for the 150 fecal samples included in the analyses, are provided as Supplementary Data S1–S3. The principal analytical workflows are summarized in Supplementary Table S4. R scripts for alpha-diversity, beta-diversity, plant family–bacterial genus association, and balanced-subsampling sensitivity analyses are provided as Supplementary Code S1.

## Supplementary Materials

The following supporting information can be downloaded at: https://www.mdpi.com/article/10.3390/biology15201847/s1: Figure S1. Multivariate dispersion of fecal microbiota and plant-derived diet among the five sampling groups; Figure S2. Comparison of unadjusted and sampling-group-adjusted associations between plant families and bacterial genera; Table S1. Pairwise geographic distances among the five sampling sites (km); Table S2. Summary of sequence processing for bacterial 16S rRNA gene V3–V4 amplicons from Common Crane fecal samples; Table S3. Summary of sequence processing for plant *trnL* amplicons from Common Crane fecal samples; Table S4. Summary of the principal analytical workflows, software/packages, and analytical procedures used in this study; Table S5. Sampling information, sequencing depths, and principal plant taxa and bacterial phyla for the five sampling groups; Table S6. Prevalence of principal bacterial and plant taxa in fecal samples across the five sampling groups; Table S7. Alpha-diversity estimates and uncertainty of fecal microbiota and plant-derived diet across the five sampling groups; Table S8. Pairwise PERMANOVA results for fecal microbiota structure among the five sampling groups; Table S9. Sensitivity of alpha-diversity comparisons to balanced subsampling; Table S10. Sensitivity of Bray–Curtis-based ANOSIM comparisons to balanced subsampling; Table S11. Pairwise PERMANOVA results for plant-derived diet composition among the five sampling groups; Table S12. Results of unadjusted and sampling-group-adjusted associations between plant families and bacterial genera. Supplementary Data S1: bacterial ASV abundance table with taxonomic assignments; Supplementary Data S2: plant *trnL* OTU abundance table with taxonomic assignments; Supplementary Data S3: non-sensitive sample metadata. Supplementary Code S1: R scripts for alpha-diversity, beta-diversity, plant family–bacterial genus association, and balanced-subsampling sensitivity analyses.

## Abbreviations

The following abbreviations are used in this manuscript:

PCR	Polymerase chain reaction
ASV	Amplicon sequence variant
OTU	Operational taxonomic unit
PCoA	Principal coordinates analysis
PERMANOVA	Permutational multivariate analysis of variance
ANOSIM	Analysis of similarities
LEfSe	Linear discriminant analysis effect size
KEGG	Kyoto Encyclopedia of Genes and Genomes
DNA	Deoxyribonucleic acid
rRNA	Ribosomal ribonucleic acid
BLAST	Basic Local Alignment Search Tool
NCBI	National Center for Biotechnology Information
SRA	Sequence Read Archive
QIIME 2	Quantitative Insights Into Microbial Ecology 2
DADA2	Divisive Amplicon Denoising Algorithm 2
LDA	Linear discriminant analysis
PERMDISP	Permutational analysis of multivariate dispersions
CLR	Centered log-ratio
FDR	False discovery rate
BH	Benjamini–Hochberg
CI	Confidence interval
PICRUSt2	Phylogenetic Investigation of Communities by Reconstruction of Unobserved States 2
