# Supplementary material for: Associations Between Plant-Derived Diet Composition and Fecal Microbiota in Wintering Common Cranes in the Yellow River Estuary
Source: Biology (Basel). 2026 Oct 15;15(20):1847. doi: 10.3390/biology15201847 (PMC13701833; doi:10.3390/biology15201847)
Supplement: Supplementary file 1 [file biology-15-01847-s001.zip › Supplementary Materials.pdf]

Figure S1.

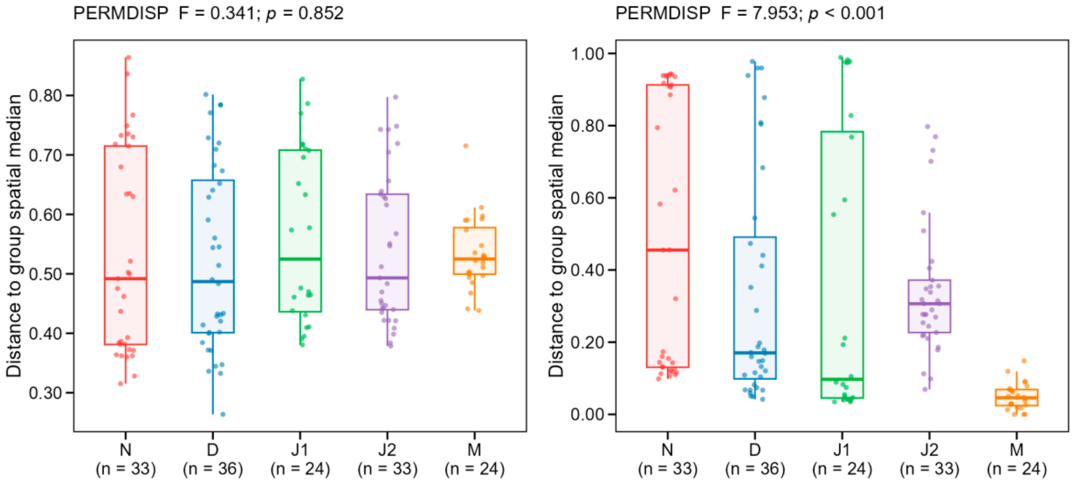

Figure S1. Multivariate dispersion of fecal microbiota and plant-derived diet among the five sampling groups. The left panel shows fecal microbiota, and the right panel shows plant-derived diet.

Figure S2.

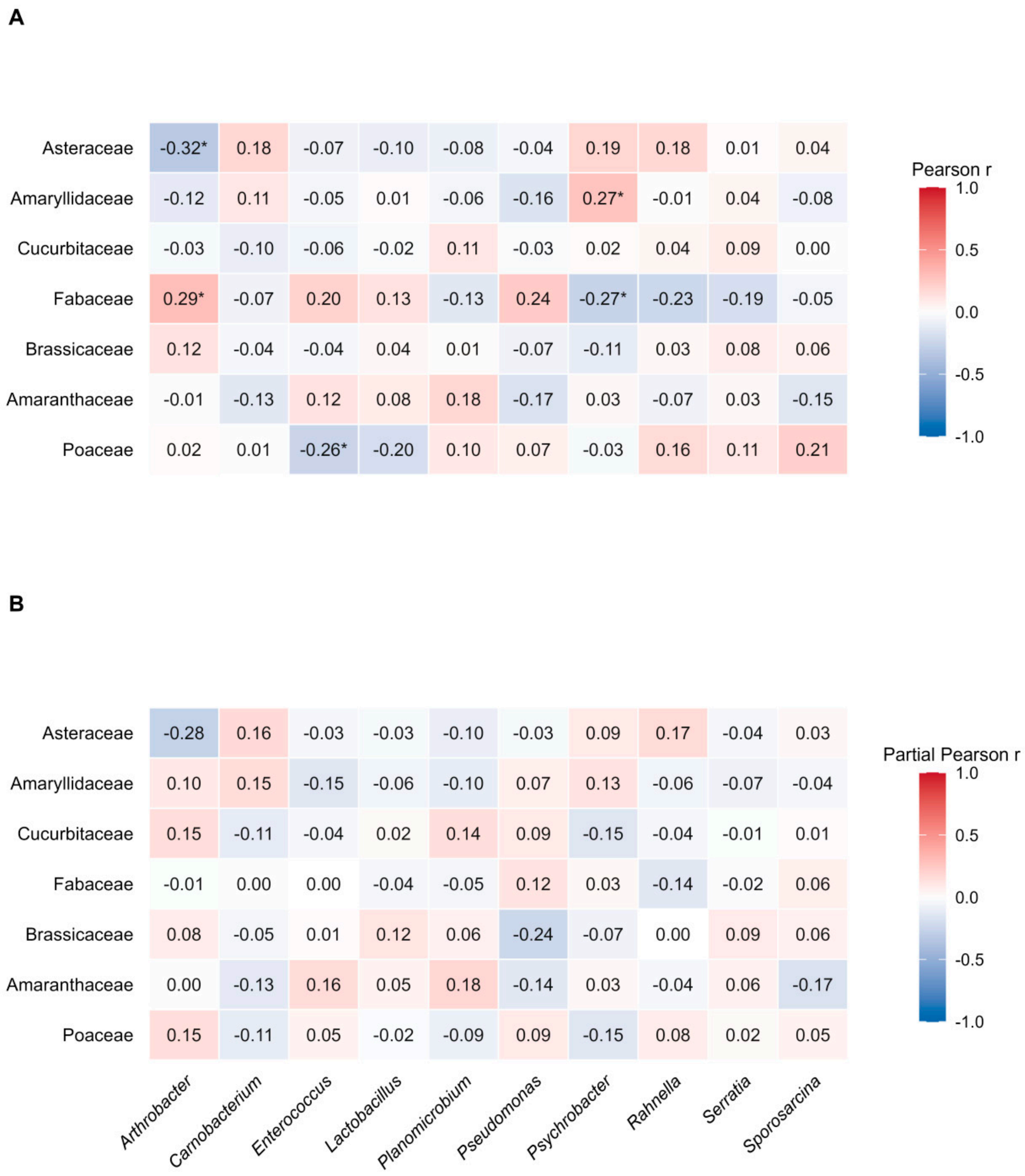

Within each panel, * denotes BH-FDR q < 0.05 across that panel's 70 tests.

Figure S2. Comparison of unadjusted and sampling-group-adjusted associations between plant families and bacterial genera. (A) Unadjusted Pearson correlations. (B) Sampling-group-adjusted partial Pearson correlations.

Table S1. Pairwise geographic distances among the five sampling sites (km).

**Sampling site**	**N**	**D**	**J1**	**J2**	**M**
N	—	39.3	27.4	3.5	36.3
D		—	12.5	35.9	3.4
J1			—	23.9	9.2
J2				—	32.8
M					—

Note: Distances were calculated from sampling-site coordinates as straight-line geographic distances. Only the upper triangle is shown because the distance matrix is symmetric.

Table S2. Summary of sequence processing for bacterial 16S rRNA gene V3–V4

amplicons from Common Crane fecal samples.

Sample	Input	Filtered	Denoised	Merged	Non-chimeric	Non-singleton
A1	99207	91333	90519	87919	75392	75311
A3	96804	89217	88514	87265	74976	74955
A5	96920	90583	89615	87066	73314	73266
A6	92388	85676	84697	81393	71672	71622
A12	87191	80231	79726	78728	65072	65059
A13	95503	88292	87847	86439	81439	81422
A14	94677	87365	86715	85712	82207	82199
A15	88961	81816	81262	79600	73053	73040
A17	97034	89703	89159	87604	80852	80832
A18	88279	81442	81111	80552	79838	79833
A19	178803	158741	157969	155962	145678	145661
A20	103051	94952	94125	91228	72762	72735
A21	95964	89213	87895	83725	75426	75334
A22	106538	98953	98461	96821	71998	71976
A23	102882	94179	93212	89678	71300	71231
A25	96367	89002	87036	81242	67342	67153
A27	114743	107021	106365	104355	91364	91338
A28	100896	93308	92708	91041	86155	86141
A29	103319	94945	94363	93132	91108	91094
A30	112899	104188	103309	99865	81746	81707
A32	105314	97219	95938	92252	73661	73554
A34	104706	97261	96565	94505	88096	88064
B1	106693	98283	97654	95834	65858	65823
B2	104471	97499	96010	90502	57128	56942
B5	96849	90520	89673	87722	78123	78087
B7	102121	94126	93466	91198	62398	62357
B8	108126	100678	99865	97450	83390	83346
B9	107147	99820	98689	95339	73582	73513
B10	89721	83540	81836	76857	54141	53987
B11	110912	101718	101065	98642	79314	79285
C2	105979	97080	95827	90397	53298	53179
D3	98564	89322	87372	79960	75506	75337
D6	104062	96503	94323	87369	68796	68542
HHH 8	93493	86242	85534	83019	68288	68265
HHH 9	94356	88349	86387	78158	52232	52138
HHH 10	96032	88384	87085	83679	81371	81269
HHH 11	95764	89443	88629	85971	60522	60459
HHH 12	92346	86385	85827	84503	78249	78238
HHH 13	87073	80432	79926	78569	70628	70606
HH 3	95843	88564	87386	82676	63125	63050
HH 4	91718	83297	82984	82537	81575	81574

Sample	Input	Filtered	Denoised	Merged	Non-chimeric	Non-singleton
HH 5	100701	91930	90250	85276	59768	59637
HH 6	97505	90764	89514	85577	77941	77818
HH 7	103998	95845	94543	90594	65599	65516
HH 8	105579	97379	96167	91939	74515	74443
HH 9	103322	94819	93751	90662	74894	74818
HH 10	99950	92846	90502	84186	61847	61616
HH 11	98189	91727	90935	86699	63256	63214
HH 12	103755	95470	94694	90263	65962	65913
HH 13	103721	95531	94995	93035	79755	79739
HH 14	102077	94236	93626	90759	61427	61390
HH 15	101728	94302	93812	92603	70553	70538
HH 16	93592	86322	85979	85731	85656	85652
HH 17	83988	77829	77027	74731	62033	62011
HI6	81046	74788	73883	70255	49725	49653
HI7	83603	77978	77028	74900	44362	44223
HI8	82432	76651	75677	71529	49653	49562
HI9	81310	74991	74332	72361	64966	64942
HI10	84094	78027	77326	74137	60819	60754
HI11	74802	69703	68987	66333	52790	52740
HI12	83692	77807	77490	76797	73864	73856
HI21	77654	71487	70529	64265	42967	42849
HI22	77372	71683	70879	68739	54753	54720
HI23	77834	72625	71679	67974	48264	48204
HI24	74878	70087	69166	64518	45320	45239
HI25	79112	72971	72047	68587	41792	41752
HI26	76844	72102	71355	67203	47225	47167
HI27	73245	67729	67036	64415	53105	53068
HI28	78421	72464	71972	70919	54715	54710
I1	98548	92288	91298	84992	58210	58099
I2	83100	76712	75846	72645	54415	54342
I3	93567	83921	82459	76357	65848	65691
I4	87462	80316	78619	72405	51862	51662
I5	97317	86546	84684	75612	56136	55847
I6	95503	89089	87937	83723	52720	52578
I7	99028	89092	87477	79633	58275	58064
I8	80887	75757	74725	71228	55964	55839
I9	98844	91034	89507	83298	64334	64230
I10	118680	109330	108206	100138	60881	60798
I11	94218	82130	80712	74647	67614	67395
I13	90103	82233	81483	77915	53036	52944
I14	97350	88825	87119	79528	61223	60920
I15	101514	88866	86818	75512	55747	55364

Sample	Input	Filtered	Denoised	Merged	Non-chimeric	Non-singleton
I16	102189	92196	90553	83029	59232	59011
I17	98307	89340	85979	75459	67143	66779
I18	86231	79965	78991	74759	52811	52713
I19	100200	92312	90667	82970	58740	58509
I20	74069	67427	66647	61544	43175	43043
I21	101346	90166	88656	79141	51725	51375
I22	90841	82590	81245	76566	54833	54712
I23	94109	85509	84009	77622	65019	64767
I24	122186	109293	107239	97052	72442	72144
I25	113522	104652	102095	90711	49361	48847
L1	113098	104901	103866	99664	92273	92203
L2	111148	100776	99725	94808	90070	90020
L3	117277	107150	106128	102424	93349	93270
L4	118035	104837	103390	96701	71371	71190
L5	121927	112423	111581	107657	99966	99914
L6	119441	108615	106335	96885	81526	81182
L7	121311	108527	106455	96424	82023	81809
L8	111902	103453	102747	99281	93115	93070
L9	122812	109310	107957	101196	82570	82389
L10	112981	101679	99965	92324	76447	76217
L11	112374	79335	78416	75206	65874	65791
L12	122320	112265	110353	102845	93581	93347
L13	114769	105762	103166	94186	82074	81643
L14	106442	96258	94851	87405	66981	66800
L15	97577	85330	83951	77114	58399	58212
L16	94237	84970	83269	75488	55751	55525
L17	112717	100948	98382	84637	67182	66734
L18	110166	100470	99399	94923	79701	79592
L19	123458	114895	113557	108050	102855	102742
L20	125522	116704	114690	104967	77451	77185
L21	120898	110479	110002	108460	99878	99806
L22	125216	115079	112754	102547	89730	89458
L23	119257	109971	109071	105241	71169	71075
L24	116078	106769	104239	91290	62892	62470
L25	121175	112668	111634	108022	90631	90531
L26	124315	112774	111842	109238	101574	101490
L27	115672	107621	105788	98206	55445	55196
L28	126211	113176	112271	107329	101545	101459
L29	116650	106367	103193	87248	72225	71773
L30	124549	113577	112443	107161	86699	86615
K2	120609	109766	108064	100363	81020	80840
K5	116425	108672	108278	107224	103396	103391

Sample	Input	Filtered	Denoised	Merged	Non-chimeric	Non-singleton
K6	118376	109296	108546	106689	101940	101910
N1	110704	101730	100658	96274	69966	69897
N2	98515	91985	91573	90350	84116	84104
N3	105166	97951	97429	96124	88075	88063
N4	97607	88912	87997	84535	61160	61095
N5	103417	95513	94614	90168	58086	57944
N6	104109	96753	96031	92053	66302	66240
N7	109056	101498	100229	95165	68754	68602
N8	113913	105376	104803	103495	89509	89495
N9	112884	104159	102971	100156	80466	80375
N10	93788	87349	86722	85468	68414	68382
N11	101999	94978	94330	92042	75274	75235
N12	111831	103974	103142	99145	70064	70004
N13	105830	97969	97260	94654	61512	61467
N14	111648	103454	101979	97210	67825	67622
N15	100555	93764	93074	90518	73728	73687
N16	104402	95684	95030	92223	68263	68216
N17	99543	91768	90513	86264	65989	65895
N18	95839	89322	88647	86346	64511	64460
N19	102064	95233	93940	88477	58463	58317
N20	100858	94045	93085	89006	61649	61564
N21	109875	101725	100944	98407	80836	80815
N22	106174	99236	98750	97190	76638	76606
N23	103449	96539	95790	91948	64518	64430
N24	106249	98943	98207	94914	65369	65291

Table S3. Summary of sequence processing for plant trnL amplicons from Common Crane fecal samples.

Sample	Input	Merged	Filtered	Non chimeric	Non singleton
A1	130389	115841	114937	103577	103534
A3	93907	90553	89383	83067	83046
A5	106211	99845	99504	98730	98712
A6	90917	84789	84040	72798	72794
A12	64614	64095	63225	62746	62746
A13	99071	90669	89714	76374	76361
A14	87331	86759	85924	85905	85895
A15	119757	108221	106976	94708	94553
A17	99786	88814	88065	75684	75667
A18	76434	76225	75507	75429	75429
A19	134808	119899	119110	97183	97169
A20	88837	53123	52257	52183	52183
A21	101589	96189	95177	87404	87390
A22	82455	72621	71832	71100	71099
A23	120961	110338	109570	99237	99166
A25	95619	91977	91215	85798	85780
A27	104288	92577	91894	84235	84227
A28	80271	79125	78325	73611	73602
A29	86242	83460	82689	76140	76137
A30	84302	82762	81965	78568	78554
A32	73776	73539	72836	72758	72758
A34	100674	90367	89613	80289	80274
B1	110757	104180	103594	103366	103363
B2	101148	83642	82815	76954	76948
B5	75274	70740	69609	67229	67226
B7	78865	77628	76651	72045	72025
B8	105256	91092	90259	79827	79794
B9	84882	81580	80707	77389	77373
B10	73750	66331	65527	58678	58676
B11	77879	69465	68625	65629	65627
C2	103380	91151	90322	86973	86934
D3	68641	68410	67658	65153	65153
D6	110231	89268	88335	77266	77171
HHH 8	112773	107547	106366	104164	104129
HHH 9	108853	103675	102734	100582	100562
HHH 10	129155	118316	117213	103429	103422
HHH 11	85343	81235	80512	73397	73376
HHH 12	83705	81218	80221	72766	72748
HHH 13	103738	74485	73652	72310	72302
HH 3	93816	90359	89533	81656	81642

Sample	Input	Merged	Filtered	Non chimeric	Non singleton
HH 4	78991	76830	76019	70959	70944
HH 5	85866	82692	81800	73117	73104
HH 6	125902	110712	109816	98548	98501
HH 7	120698	109964	108907	101612	101526
HH 8	85386	83562	82309	79420	79404
HH 9	100841	97576	96400	87352	87328
HH 10	103660	99646	98924	95662	95639
HH 11	105986	101680	100730	91927	91901
HH 12	127574	117320	116409	104530	104414
HH 13	101681	92295	91149	88675	88675
HH 14	104113	99907	98869	93258	93230
HH 15	106951	92328	91575	79230	79208
HH 16	82237	81382	80526	73803	73801
HH 17	99776	89632	88922	80329	80286
HI6	72722	71385	70554	64984	64962
HI7	109775	106829	105714	99105	99093
HI8	117935	111422	110110	102314	102283
HI9	109204	104542	103437	97773	97729
HI10	121897	114087	113120	102027	101970
HI11	100856	100486	99341	95136	95133
HI12	90479	83603	82736	73869	73862
HI21	111547	102609	101389	98241	98197
HI22	93552	87499	86610	77223	77213
HI23	97430	78706	77901	73725	73695
HI24	100135	92731	91828	82549	82537
HI25	137060	120979	119891	106808	106745
HI26	80096	79264	78973	78623	78609
HI27	106388	102154	100882	98596	98556
HI28	109321	105332	104367	101626	101564
I1	94987	93631	92708	89294	89273
I2	127546	118268	117095	108037	107924
I3	103718	99155	98228	92686	92603
I4	93600	91544	90456	87557	87531
I5	112932	106632	105525	98490	98417
I6	87868	82874	81986	79963	79945
I7	105588	102782	101819	93392	93333
I8	102214	99622	98553	89813	89737
I9	131527	114018	112664	101507	101435
I10	318661	300201	299013	292061	291966
I11	75181	74022	73339	71214	71188
I13	82636	81998	81201	79815	79805
I14	84966	83154	82570	79463	79409

Sample	Input	Merged	Filtered	Non chimeric	Non singleton
I15	85235	83103	82548	75769	75728
I16	91499	89844	89247	83855	83806
I17	78480	75398	74804	69854	69836
I18	91031	87873	87001	84441	84404
I19	80462	78256	77677	76775	76742
I20	79678	69386	68799	67415	67392
I21	105839	101246	100616	96345	96233
I22	82498	80573	79929	79414	79384
I23	106334	104871	103965	99907	99878
I24	86284	85309	84344	79000	78976
I25	121191	117438	115854	103215	103070
L1	98877	98442	97288	90787	90779
L2	105383	104718	103519	91013	91007
L3	106300	101984	100771	91218	91210
L4	109771	103050	101860	93976	93963
L5	113262	108049	106519	98313	98287
L6	85284	75281	74355	74328	74328
L7	104169	95329	94219	90433	90433
L8	92165	91031	89907	80557	80536
L9	106153	96279	94693	88709	88695
L10	98587	98107	97164	85451	85431
L11	95911	86023	84902	78263	78260
L12	93079	91878	90845	79172	79160
L13	109576	109275	108475	108129	108119
L14	96221	92099	91254	83128	83111
L15	94669	93486	92631	84988	84969
L16	103877	101002	99932	90692	90638
L17	84894	83639	82660	82453	82451
L18	97402	95676	94765	89721	89707
L19	92822	85097	84349	77256	77254
L20	89742	89284	88346	80540	80528
L21	103945	103234	102012	93196	93184
L22	124874	121201	119738	114615	114585
L23	124249	122575	120497	114116	114098
L24	125095	117215	115714	104269	104242
L25	116316	115854	114279	101983	101981
L26	137735	137084	135492	127397	127390
L27	133078	132555	131109	131000	130998
L28	142733	142083	140052	125238	125221
L29	131948	128790	127015	113181	113175
L30	123636	122993	121891	121831	121829
K2	136333	122970	121722	117846	117844

Sample	Input	Merged	Filtered	Non chimeric	Non singleton
K5	157674	148395	147092	144065	144034
K6	126435	120345	118827	113174	113169
N1	141608	140849	140429	140095	140088
N2	146837	137226	136759	135893	135875
N3	142302	141434	140943	140290	140282
N4	148649	147448	146886	145413	145401
N5	137879	137022	136632	136302	136296
N6	142975	142059	141511	141328	141317
N7	141686	140732	140164	139331	139323
N8	141622	140759	140279	140151	140146
N9	144226	143384	142968	141557	141520
N10	147382	146085	145594	143924	143908
N11	136125	135179	134686	133185	133161
N12	143319	139696	139223	138908	138895
N13	141450	134918	134592	126935	126855
N14	131191	130077	129700	128358	128322
N15	137836	136395	135939	133827	133806
N16	147483	146562	146046	145889	145884
N17	134548	133429	133035	132196	132185
N18	134622	117176	116779	115998	115988
N19	143960	140833	140336	139057	139014
N20	138032	136793	136400	135462	135443
N21	134654	120119	119716	119264	119257
N22	132500	119137	118720	117595	117583
N23	147185	146177	145628	144714	144698
N24	145150	143321	142889	139066	139035

Table S4. Summary of the principal analytical workflows, software/packages, and analytical procedures used in this study.

Analysis	Main software/packages	Main analytical procedures
Alpha-diversity analysis	R; GenesCloud	Chao1, Observed species, Shannon, and Simpson indices were calculated from the final abundance tables in R. Differences among sampling groups were evaluated using the Kruskal–Wallis test followed by Dunn's post hoc comparisons. The resulting alpha-diversity data were subsequently visualized using a bioinformatics analysis platform.
Beta-diversity analysis	R; vegan; permute	Bray–Curtis dissimilarities were used for principal coordinate analysis (PCoA), PERMANOVA, ANOSIM, and PERMDISP. Overall and pairwise PERMANOVA tests were performed using 9,999 permutations, with Benjamini–Hochberg correction applied to pairwise comparisons.
LEfSe	LEfSe; GenesCloud	LEfSe was used to identify differentially abundant bacterial taxa among sampling groups by combining non-parametric statistical testing with linear discriminant analysis (LDA). An LDA score threshold of 4.0 was used.
Correlation analysis	R; base R and stats functions	Plant-family and bacterial-genus abundance data were subjected to multiplicative zero replacement and centered log-ratio (CLR) transformation. Pearson correlations and sampling-group-adjusted residual correlations were evaluated using permutation tests, with Benjamini–Hochberg false discovery rate correction applied across 70 taxon-pair tests.
Microbial functional prediction and comparison	PICRUSt2; KEGG; GenesCloud	Microbial functional profiles were predicted using PICRUSt2 and annotated based on the KEGG database, with subsequent analyses conducted at KEGG Level 2. Differences among sampling groups were evaluated using the Kruskal–Wallis test followed by Dunn's post hoc comparisons and Benjamini–Hochberg correction.

Note: Complete R scripts for the alpha-diversity, beta-diversity, and correlation analyses are provided in Supplementary Code S1. Selected bioinformatics analyses and visualizations were performed using the GenesCloud platform (https://www.genescloud.cn).

Table S5. Sampling information, sequencing depths, and principal plant taxa and bacterial phyla for the five sampling groups.

A. Sampling information and mean sequencing depths

Group	Sampling date	Locality	Habitat	Sample size	Mean 16S rRNA depth (reads/sample)	Mean trnL depth (reads/sample)
N	November 2025	Near the former Yellow River channel	Farmland	33	76,482.6	79,314.1
D	December 2025	Near the current Yellow River channel	Farmland	36	62,259.5	88,285.8
J1	January 2026	Near the current Yellow River channel	Farmland	24	57,736.4	94,917.3
J2	January 2026	Near the former Yellow River channel	Farmland	33	82,571.2	98,806.5
M	March 2026	Near the current Yellow River channel	Farmland	24	70,325.2	135,595.1

B. Principal plant taxa and bacterial phyla

Group	Plant taxa (relative read abundance, %)	Bacterial phyla (relative read abundance, %)
N	*Triticum aestivum* (63.12)<br>*Zea mays* (11.08)<br>Asteraceae (8.88)	Firmicutes (70.73)<br>Proteobacteria (18.16)<br>Actinobacteria (3.75)
D	*Triticum aestivum* (88.43)<br>*Sorghum bicolor* (4.79)<br>*Zea mays* (3.90)	Firmicutes (79.61)<br>Proteobacteria (13.32)<br>Actinobacteria (4.89)
J1	*Sorghum bicolor* (72.98)<br>*Oryza sativa* (11.42)<br>*Zea mays* (6.34)	Firmicutes (57.15)<br>Proteobacteria (29.80)<br>Actinobacteria (11.20)
J2	*Triticum aestivum* (57.27)<br>*Glycine max* (26.07)<br>*Zea mays* (9.03)	Firmicutes (54.49)<br>Proteobacteria (31.89)<br>Actinobacteria (10.62)
M	*Oryza sativa* (100.00)	Proteobacteria (74.90)<br>Firmicutes (20.52)<br>Actinobacteria (3.26)

Note: Depths are based on non-singleton reads. Up to three principal named taxa per group are shown; “Others” is excluded.

Table S6. Prevalence of principal bacterial and plant taxa in fecal samples across the five sampling groups.

A. Bacterial taxa

Phylum level

Taxon	N(n = 33)	D(n = 36)	J1(n = 24)	J2(n = 33)	M(n = 24)
Firmicutes	33 (100.0)	36 (100.0)	24 (100.0)	33 (100.0)	24 (100.0)
Proteobacteria	33 (100.0)	36 (100.0)	24 (100.0)	33 (100.0)	24 (100.0)
Actinobacteria	33 (100.0)	35 (97.2)	24 (100.0)	33 (100.0)	24 (100.0)
Fusobacteria	19 (57.6)	18 (50.0)	6 (25.0)	9 (27.3)	8 (33.3)
Bacteroidetes	33 (100.0)	31 (86.1)	18 (75.0)	30 (90.9)	22 (91.7)
Tenericutes	11 (33.3)	9 (25.0)	1 (4.2)	5 (15.2)	2 (8.3)
Cyanobacteria	29 (87.9)	27 (75.0)	24 (100.0)	33 (100.0)	16 (66.7)
Chloroflexi	28 (84.8)	24 (66.7)	23 (95.8)	33 (100.0)	18 (75.0)
Acidobacteria	27 (81.8)	21 (58.3)	12 (50.0)	29 (87.9)	9 (37.5)
Gemmatimonadetes	28 (84.8)	25 (69.4)	16 (66.7)	31 (93.9)	4 (16.7)

Genus level

Taxon	N(n = 33)	D(n = 36)	J1(n = 24)	J2(n = 33)	M(n = 24)
Lactobacillus	33 (100.0)	36 (100.0)	24 (100.0)	33 (100.0)	24 (100.0)
Pseudomonas	30 (90.9)	33 (91.7)	24 (100.0)	33 (100.0)	24 (100.0)
Psychrobacter	33 (100.0)	26 (72.2)	23 (95.8)	26 (78.8)	24 (100.0)
Planomicrobium	24 (72.7)	36 (100.0)	22 (91.7)	32 (97.0)	24 (100.0)
Arthrobacter	27 (81.8)	35 (97.2)	24 (100.0)	32 (97.0)	24 (100.0)
Sporosarcina	20 (60.6)	30 (83.3)	23 (95.8)	25 (75.8)	23 (95.8)
Enterococcus	33 (100.0)	26 (72.2)	22 (91.7)	32 (97.0)	9 (37.5)
Carnobacterium	12 (36.4)	23 (63.9)	22 (91.7)	18 (54.5)	23 (95.8)
Rahnella	4 (12.1)	3 (8.3)	11 (45.8)	9 (27.3)	23 (95.8)
Serratia	4 (12.1)	2 (5.6)	4 (16.7)	3 (9.1)	22 (91.7)

Note: Values are numbers of samples with relative abundance >0, with percentages in parentheses.

B. Plant taxa

Plant taxon	N (n = 33)	D (n = 36)	J1 (n = 24)	J2 (n = 33)	M (n = 24)
Triticum aestivum	33 (100.0)	36 (100.0)	24 (100.0)	33 (100.0)	0 (0.0)
Zea mays	31 (93.9)	36 (100.0)	24 (100.0)	33 (100.0)	9 (37.5)
Asteraceae	14 (42.4)	12 (33.3)	15 (62.5)	10 (30.3)	11 (45.8)
Allium sativum	13 (39.4)	4 (11.1)	0 (0.0)	1 (3.0)	0 (0.0)
Sorghum bicolor	20 (60.6)	33 (91.7)	24 (100.0)	25 (75.8)	1 (4.2)
Poaceae	21 (63.6)	25 (69.4)	5 (20.8)	20 (60.6)	0 (0.0)
Cucurbitaceae	9 (27.3)	1 (2.8)	3 (12.5)	4 (12.1)	1 (4.2)
Oryza sativa	7 (21.2)	26 (72.2)	24 (100.0)	18 (54.5)	24 (100.0)
Glycine max	22 (66.7)	17 (47.2)	22 (91.7)	32 (97.0)	1 (4.2)
Brassicaceae	4 (12.1)	0 (0.0)	5 (20.8)	17 (51.5)	0 (0.0)
Suaeda salsa	9 (27.3)	9 (25.0)	10 (41.7)	13 (39.4)	1 (4.2)

Note: Values represent the number of samples in which each plant taxon was detected, with the corresponding percentages in parentheses. Detection was defined as a non-zero read count in the sample-level plant OTU abundance table.

Table S7. Alpha-diversity estimates and uncertainty of fecal microbiota and plant-derived diet across the five sampling groups.

A. Fecal microbiota

Index	Group	n	Median（Q1–Q3）	Bootstrap 95% CI of the median
Chao1	N	33	420.6 (308.2–584.5)	338.0–498.4
	D	36	515.0 (361.9–709.8)	422.7–679.0
	J1	24	1393.5 (764.7–1558.3)	834.7–1552.0
	J2	33	1076.3 (656.7–1461.8)	858.9–1137.7
	M	24	576.5 (451.8–671.2)	461.6–652.9
Simpson	N	33	0.810 (0.678–0.867)	0.682–0.845
	D	36	0.784 (0.675–0.886)	0.729–0.877
	J1	24	0.905 (0.847–0.941)	0.870–0.932
	J2	33	0.854 (0.767–0.907)	0.792–0.885
	M	24	0.924 (0.880–0.947)	0.881–0.944
Shannon	N	33	3.732 (3.083–4.370)	3.317–4.146
	D	36	3.984 (3.335–5.013)	3.695–4.821
	J1	24	5.347 (5.035–6.267)	5.082–5.936
	J2	33	4.884 (3.915–5.584)	4.535–5.240
	M	24	5.268 (4.586–5.687)	4.692–5.534
Observed species	N	33	385.0 (289.0–545.0)	297.0–428.0
	D	36	467.0 (326.0–658.2)	381.0–588.0
	J1	24	1155.5 (692.2–1373.0)	734.0–1328.0
	J2	33	930.0 (599.0–1292.0)	812.0–1044.0
	M	24	499.0 (384.5–586.2)	423.0–566.5

B. Plant-derived diet

Index	Group	n	Median（Q1–Q3）	Bootstrap 95% CI of the median
Chao1	N	33	6.00 (5.00–7.00)	5.00–6.00
	D	36	6.00 (5.00–6.12)	5.00–6.00
	J1	24	6.00 (6.00–7.00)	6.00–7.00
	J2	33	7.00 (5.00–7.00)	6.00–7.00
	M	24	2.00 (1.00–3.00)	1.50–2.50
Simpson	N	33	0.054 (0.008–0.406)	0.020–0.242
	D	36	0.066 (0.025–0.316)	0.033–0.215
	J1	24	0.033 (0.013–0.234)	0.016–0.137
	J2	33	0.432 (0.335–0.485)	0.368–0.467
	M	24	0.001 (0.000–0.010)	0.000–0.008
Shannon	N	33	0.132 (0.028–0.663)	0.038–0.413
	D	36	0.177 (0.073–0.633)	0.093–0.462
	J1	24	0.095 (0.047–0.462)	0.049–0.330
	J2	33	0.674 (0.551–0.719)	0.629–0.693
	M	24	0.005 (0.000–0.032)	0.000–0.027
Observed species	N	33	5.00 (4.00–6.00)	5.00–6.00
	D	36	6.00 (5.00–6.00)	5.00–6.00
	J1	24	6.00 (6.00–7.00)	6.00–7.00
	J2	33	7.00 (5.00–7.00)	6.00–7.00
	M	24	2.00 (1.00–3.00)	2.00–2.50

Note: Values are presented as median (Q1–Q3). The 95% CIs are percentile confidence intervals for the median obtained from 10,000 within-group nonparametric bootstrap resamples.

Table S8. Pairwise PERMANOVA results for fecal microbiota structure among the five sampling groups.

Group 1	Group 2	n_1	n_2	df	pseudo-F	R^2	p	p_{adj}
N	D	33	36	1, 67	5.169	0.0716	0.0001	0.0001
N	J1	33	24	1, 55	5.999	0.0983	0.0001	0.0001
N	J2	33	33	1, 64	4.067	0.0598	0.0001	0.0001
N	M	33	24	1, 55	14.054	0.2035	0.0001	0.0001
D	J1	36	24	1, 58	4.154	0.0668	0.0004	0.0004
D	J2	36	33	1, 67	5.674	0.0781	0.0001	0.0001
D	M	36	24	1, 58	13.257	0.1860	0.0001	0.0001
J1	J2	24	33	1, 55	4.547	0.0764	0.0001	0.0001
J1	M	24	24	1, 46	7.145	0.1344	0.0001	0.0001
J2	M	33	24	1, 55	13.326	0.1950	0.0001	0.0001

Note: Pairwise PERMANOVA was performed using Bray–Curtis dissimilarities with 9,999 unrestricted permutations. df denotes between-group and residual degrees of freedom; p_{adj} denotes the Benjamini–Hochberg-adjusted p-value across the 10 comparisons within this dataset.

Table S9. Sensitivity of alpha-diversity comparisons to balanced subsampling.

A1. Overall comparisons

Dataset	Index	Full-data H	Full-data p	Significant iterations (%)
Fecal microbiota	Chao1	55.013	<0.001	100.0
Fecal microbiota	Observed richness	54.973	<0.001	100.0
Fecal microbiota	Shannon	41.958	<0.001	100.0
Fecal microbiota	Simpson	32.671	<0.001	100.0
Plant-derived diet	Chao1	64.047	<0.001	100.0
Plant-derived diet	Observed richness	68.981	<0.001	100.0
Plant-derived diet	Shannon	53.606	<0.001	100.0
Plant-derived diet	Simpson	54.750	<0.001	100.0

A2. Pairwise comparisons: fecal microbiota

Index	Comparison	Full-data BH-adjusted p	Median difference under subsampling (2.5th–97.5th percentiles)	Direction agreement (%)	Significant iterations (%)
Chao1	N–D	0.491	-99.03 (-203.16–-23.78)	99.5	0.0
Chao1	N–J1	<0.001	-981.77 (-1033.38–-913.59)	100.0	100.0

Index	Comparison	Full-data BH-adjusted p	Median difference under subsampling (2.5th–97.5th percentiles)	Direction agreement (%)	Significant iterations (%)
Chao1	N–J2	<0.001	-648.80 (-725.09–-543.15)	100.0	100.0
Chao1	N–M	0.344	-164.74 (-216.35–-96.55)	100.0	0.1
Chao1	D–J1	<0.001	-878.49 (-932.09–-764.72)	100.0	100.0
Chao1	D–J2	<0.001	-558.77 (-623.89–-427.88)	100.0	100.0
Chao1	D–M	0.624	-61.45 (-115.05–52.31)	94.7	0.0
Chao1	J1–J2	0.491	312.73 (267.56–425.55)	100.0	0.0
Chao1	J1–M	<0.001	817.04 (817.04–817.04)	100.0	100.0
Chao1	J2–M	<0.001	504.31 (391.49–549.47)	100.0	100.0
Observed richness	N–D	0.463	-88.00 (-184.00–-18.50)	99.4	0.0
Observed richness	N–J1	<0.001	-780.50 (-836.00–-732.00)	100.0	100.0
Observed richness	N–J2	<0.001	-545.50 (-647.00–-433.00)	100.0	100.0
Observed richness	N–M	0.367	-124.00 (-179.50–-75.50)	100.0	0.0
Observed richness	D–J1	<0.001	-688.50 (-742.00–-579.50)	100.0	100.0

Index	Comparison	Full-data BH-adjusted p	Median difference under subsampling (2.5th–97.5th percentiles)	Direction agreement (%)	Significant iterations (%)
Observed richness	D–J2	<0.001	-460.00 (-556.00–-345.50)	100.0	99.9
Observed richness	D–M	0.739	-32.00 (-85.50–77.00)	86.9	0.0
Observed richness	J1–J2	0.367	222.50 (135.00–338.00)	100.0	0.0
Observed richness	J1–M	<0.001	656.50 (656.50–656.50)	100.0	100.0
Observed richness	J2–M	<0.001	434.00 (318.50–521.50)	100.0	100.0
Shannon	N–D	0.322	-0.242 (-0.618–0.130)	86.8	0.3
Shannon	N–J1	<0.001	-1.559 (-1.856–-1.284)	100.0	100.0
Shannon	N–J2	0.001	-1.091 (-1.421–-0.753)	100.0	95.3
Shannon	N–M	<0.001	-1.480 (-1.777–-1.205)	100.0	100.0
Shannon	D–J1	<0.001	-1.363 (-1.537–-1.065)	100.0	100.0
Shannon	D–J2	0.023	-0.845 (-1.152–-0.523)	100.0	44.5
Shannon	D–M	0.001	-1.284 (-1.458–-0.986)	100.0	100.0
Shannon	J1–J2	0.036	0.496 (0.273–0.657)	100.0	41.8
Shannon	J1–M	0.322	0.079 (0.079–0.079)	100.0	0.0

Index	Comparison	Full-data BH-adjusted p	Median difference under subsampling (2.5th–97.5th percentiles)	Direction agreement (%)	Significant iterations (%)
Shannon	J2–M	0.301	-0.417 (-0.579–-0.194)	100.0	0.1
Simpson	N–D	0.843	0.0191 (-0.0828–0.0863)	67.0	0.0
Simpson	N–J1	0.001	-0.1005 (-0.1689–-0.0732)	100.0	100.0
Simpson	N–J2	0.116	-0.0493 (-0.1226–-0.0026)	98.1	7.2
Simpson	N–M	<0.001	-0.1196 (-0.1879–-0.0922)	100.0	100.0
Simpson	D–J1	0.001	-0.1202 (-0.1709–-0.0346)	100.0	99.9
Simpson	D–J2	0.142	-0.0732 (-0.1252–0.0198)	90.8	4.5
Simpson	D–M	<0.001	-0.1393 (-0.1900–-0.0537)	100.0	100.0
Simpson	J1–J2	0.073	0.0463 (0.0305–0.0911)	100.0	18.6
Simpson	J1–M	0.462	-0.0191 (-0.0191–-0.0191)	100.0	0.0
Simpson	J2–M	0.008	-0.0654 (-0.1102–-0.0496)	100.0	91.5

A3. Pairwise comparisons: plant-derived diet

Index	Comparison	Full-data BH-adjusted p	Median difference under subsampling (2.5th–97.5th percentiles)	Direction agreement (%)	Significant iterations (%)
Chao1	N–D	0.860	0.00 (-1.00–0.00)	NA	0.0

Index	Comparison	Full-data BH-adjusted p	Median difference under subsampling (2.5th–97.5th percentiles)	Direction agreement (%)	Significant iterations (%)
Chao1	N–J1	0.271	0.00 (-1.00–0.00)	NA	0.4
Chao1	N–J2	0.206	-1.00 (-2.00–-1.00)	100.0	1.4
Chao1	N–M	<0.001	4.00 (3.00–4.00)	100.0	100.0
Chao1	D–J1	0.206	0.00 (0.00–0.25)	NA	0.2
Chao1	D–J2	0.151	-1.00 (-1.00–-0.75)	100.0	2.8
Chao1	D–M	<0.001	4.00 (4.00–4.25)	100.0	100.0
Chao1	J1–J2	0.917	-1.00 (-1.00–-1.00)	100.0	0.0
Chao1	J1–M	<0.001	4.00 (4.00–4.00)	100.0	100.0
Chao1	J2–M	<0.001	5.00 (5.00–5.00)	100.0	100.0
Observed richness	N–D	0.826	-0.50 (-1.00–0.50)	67.8	0.0
Observed richness	N–J1	0.036	-0.50 (-1.00–0.00)	74.2	36.0
Observed richness	N–J2	0.072	-1.00 (-2.00–0.00)	91.9	10.7
Observed richness	N–M	<0.001	3.50 (3.00–4.00)	100.0	100.0
Observed richness	D–J1	0.045	0.00 (-1.00–0.00)	NA	19.7
Observed richness	D–J2	0.094	-0.50 (-1.50–0.00)	77.5	5.3
Observed richness	D–M	<0.001	4.00 (3.00–4.00)	100.0	100.0

Index	Comparison	Full-data BH-adjusted p	Median difference under subsampling (2.5th–97.5th percentiles)	Direction agreement (%)	Significant iterations (%)
Observed richness	J1–J2	0.631	-0.50 (-1.00–0.00)	73.1	0.0
Observed richness	J1–M	<0.001	4.00 (4.00–4.00)	100.0	100.0
Observed richness	J2–M	<0.001	4.50 (4.00–5.00)	100.0	100.0
Shannon	N–D	0.543	-0.051 (-0.277–0.122)	77.3	0.2
Shannon	N–J1	0.847	0.041 (-0.025–0.212)	89.3	0.0
Shannon	N–J2	0.005	-0.532 (-0.598–-0.370)	100.0	75.2
Shannon	N–M	<0.001	0.131 (0.065–0.302)	100.0	100.0
Shannon	D–J1	0.508	0.082 (0.031–0.301)	99.7	0.0
Shannon	D–J2	0.024	-0.491 (-0.550–-0.257)	100.0	28.6
Shannon	D–M	<0.001	0.172 (0.121–0.391)	100.0	100.0
Shannon	J1–J2	0.005	-0.575 (-0.593–-0.538)	100.0	92.5
Shannon	J1–M	<0.001	0.090 (0.090–0.090)	100.0	100.0
Shannon	J2–M	<0.001	0.665 (0.627–0.683)	100.0	100.0
Simpson	N–D	0.602	-0.0154 (-0.1327–0.0726)	64.8	0.1

Index	Comparison	Full-data BH-adjusted p	Median difference under subsampling (2.5th–97.5th percentiles)	Direction agreement (%)	Significant iterations (%)
Simpson	N–J1	0.791	0.0246 (-0.0089–0.1187)	91.7	0.0
Simpson	N–J2	0.002	-0.3819 (-0.4242–-0.2893)	100.0	85.7
Simpson	N–M	<0.001	0.0560 (0.0225–0.1501)	100.0	100.0
Simpson	D–J1	0.510	0.0335 (0.0103–0.1484)	99.7	0.0
Simpson	D–J2	0.010	-0.3641 (-0.4109–-0.2492)	100.0	49.8
Simpson	D–M	<0.001	0.0649 (0.0416–0.1798)	100.0	100.0
Simpson	J1–J2	0.002	-0.4059 (-0.4336–-0.3814)	100.0	99.8
Simpson	J1–M	<0.001	0.0314 (0.0314–0.0314)	100.0	100.0
Simpson	J2–M	<0.001	0.4373 (0.4128–0.4650)	100.0	100.0

Notes. Original sample sizes were N = 33, D = 36, J1 = 24, J2 = 33, and M = 24. In each of 1,000 iterations, 24 samples per group were analyzed by subsampling N, D, and J2 without replacement and retaining all J1 and M samples. Overall comparisons used Kruskal–Wallis tests; pairwise comparisons used two-sided Dunn tests with BH correction across the 10 comparisons per dataset and index. Median differences are the first group minus the second. Direction agreement indicates consistency with the full-data difference; NA denotes a zero full-data median difference. Significant iterations (%) reports the percentage with $p < 0.05$ overall or BH-adjusted $p < 0.05$ pairwise. Percentile ranges describe subsampling variability, not confidence intervals; percentages indicate stability under this sampling scheme, not statistical power or new p-values.

Table S10. Sensitivity of Bray–Curtis-based ANOSIM comparisons to balanced subsampling.

B1. Overall comparisons

Dataset	Full-data R	Full-data p	Subsampling R, median (2.5th–97.5th percentiles)	Significant iterations (%)
Fecal microbiota	0.3363	<0.001	0.3638 (0.3417–0.3894)	100.0
Plant-derived diet	0.5500	<0.001	0.6333 (0.6176–0.6531)	100.0

B2. Pairwise comparisons

Dataset	Comparison	Full-data R	Full-data BH-adjusted p	Subsampling R, median (2.5th–97.5th percentiles)	Significant iterations (%)
Fecal microbiota	N–D	0.1689	<0.001	0.1688 (0.1170–0.2323)	100.0
Fecal microbiota	N–J1	0.2800	<0.001	0.2627 (0.2420–0.3025)	100.0
Fecal microbiota	N–J2	0.1468	<0.001	0.1483 (0.1084–0.2045)	100.0
Fecal microbiota	N–M	0.7089	<0.001	0.7501 (0.7076–0.8408)	100.0
Fecal microbiota	D–J1	0.1715	<0.001	0.1326 (0.1050–0.1725)	100.0
Fecal microbiota	D–J2	0.1973	<0.001	0.1992 (0.1495–0.2600)	100.0
Fecal microbiota	D–M	0.6205	<0.001	0.6800 (0.6051–0.7695)	100.0
Fecal microbiota	J1–J2	0.2249	<0.001	0.1945 (0.1709–0.2231)	100.0
Fecal microbiota	J1–M	0.4702	<0.001	0.4702 (fixed)	100.0[a]
Fecal microbiota	J2–M	0.7646	<0.001	0.7955 (0.7595–0.8480)	100.0

Dataset	Comparison	Full-data R	Full-data BH-adjusted p	Subsampling R, median (2.5th–97.5th percentiles)	Significant iterations (%)
Plant-derived diet	N–D	0.0435	0.018	0.0236 (-0.0003–0.0637)	27.6
Plant-derived diet	N–J1	0.4782	<0.001	0.5295 (0.4598–0.6166)	100.0
Plant-derived diet	N–J2	0.0931	<0.001	0.0934 (0.0607–0.1301)	100.0
Plant-derived diet	N–M	0.9129	<0.001	0.9340 (0.9085–0.9606)	100.0
Plant-derived diet	D–J1	0.6419	<0.001	0.6201 (0.5439–0.6994)	100.0
Plant-derived diet	D–J2	0.0954	<0.001	0.0968 (0.0541–0.1473)	100.0
Plant-derived diet	D–M	0.9206	<0.001	0.9433 (0.9012–0.9822)	100.0
Plant-derived diet	J1–J2	0.7979	<0.001	0.7730 (0.7406–0.8040)	100.0
Plant-derived diet	J1–M	0.8424	<0.001	0.8424 (fixed)	100.0[a]
Plant-derived diet	J2–M	0.9873	<0.001	0.9908 (0.9830–0.9985)	100.0

Notes. Balanced subsampling followed Table S9. ANOSIM used Bray–Curtis distances and 9,999 unrestricted permutations. Significant iterations (%) reports the percentage with $p < 0.05$ overall or BH-adjusted $p < 0.05$ across the 10 pairwise comparisons per dataset. Superscript a: J1 and M each contained 24 samples, so their comparison used identical samples throughout; R and the raw permutation p-value were calculated once and reused, while the BH-adjusted p-value could vary. Percentile ranges describe subsampling variability, not confidence intervals; percentages indicate stability under this sampling scheme, not statistical power or new p-values.

Table S11. Pairwise PERMANOVA results for plant-derived diet composition among the five sampling groups.

Group 1	Group 2	n_1	n_2	df	pseudo-F	R^2	p	p_{adj}
N	D	33	36	1, 67	2.516	0.0362	0.0266	0.0266
N	J1	33	24	1, 55	17.162	0.2378	0.0001	0.0001
N	J2	33	33	1, 64	5.101	0.0738	0.0003	0.0003
N	M	33	24	1, 55	50.077	0.4766	0.0001	0.0001
D	J1	36	24	1, 58	31.213	0.3499	0.0001	0.0001
D	J2	36	33	1, 67	5.456	0.0753	0.0002	0.0003
D	M	36	24	1, 58	95.222	0.6215	0.0001	0.0001
J1	J2	24	33	1, 55	39.694	0.4192	0.0001	0.0001
J1	M	24	24	1, 46	64.842	0.5850	0.0001	0.0001
J2	M	33	24	1, 55	133.100	0.7076	0.0001	0.0001

Note: Pairwise PERMANOVA was performed using Bray–Curtis dissimilarities with 9,999 unrestricted permutations. df denotes between-group and residual degrees of freedom; p_{adj} denotes the Benjamini–Hochberg-adjusted p-value across the 10 comparisons within this dataset. Multivariate dispersion differed significantly among sampling groups for the plant-derived diet dataset; therefore, the corresponding PERMANOVA results should be interpreted with consideration of this dispersion heterogeneity.

Table S12. Results of unadjusted and sampling-group-adjusted associations between plant families and bacterial genera.

Plant family	Bacterial genus	Unadjusted analysis			Sampling-group-adjusted analysis		
		Pearson *r*	Permutation *p*	BH-FDR *q*	Partial Pearson *r*	Permutation *p*	BH-FDR *q*
Asteraceae	*Arthrobacter*	-0.325	0.0002	0.0140	-0.279	0.0011	0.0770
Asteraceae	*Carnobacterium*	0.181	0.0297	0.1386	0.164	0.0432	0.4125
Asteraceae	*Enterococcus*	-0.071	0.3890	0.7105	-0.035	0.6861	0.8739
Asteraceae	*Lactobacillus*	-0.096	0.2503	0.5475	-0.028	0.7241	0.8739
Asteraceae	*Planomicrobium*	-0.081	0.3241	0.6442	-0.097	0.2323	0.6254
Asteraceae	*Pseudomonas*	-0.044	0.6007	0.8347	-0.031	0.6969	0.8739
Asteraceae	*Psychrobacter*	0.191	0.0171	0.1088	0.092	0.2943	0.6867
Asteraceae	*Rahnella*	0.177	0.0293	0.1386	0.165	0.0354	0.4125
Asteraceae	*Serratia*	0.008	0.9236	0.9617	-0.044	0.5441	0.8168
Asteraceae	*Sporosarcina*	0.044	0.5915	0.8347	0.027	0.7206	0.8739
Amaryllidaceae	*Arthrobacter*	-0.118	0.1463	0.4096	0.096	0.3165	0.6923
Amaryllidaceae	*Carnobacterium*	0.110	0.1801	0.4587	0.154	0.0426	0.4125
Amaryllidaceae	*Enterococcus*	-0.045	0.5944	0.8347	-0.154	0.0766	0.4125
Amaryllidaceae	*Lactobacillus*	0.007	0.9272	0.9617	-0.060	0.4138	0.7427
Amaryllidaceae	*Planomicrobium*	-0.057	0.4897	0.7618	-0.104	0.1466	0.5073
Amaryllidaceae	*Pseudomonas*	-0.156	0.0606	0.2357	0.069	0.3667	0.7334
Amaryllidaceae	*Psychrobacter*	0.268	0.0006	0.0140	0.125	0.1664	0.5295
Amaryllidaceae	*Rahnella*	-0.006	0.9427	0.9617	-0.058	0.3601	0.7334

Plant family	Bacterial genus	Unadjusted analysis			Sampling-group-adjusted analysis		
		Pearson *r*	Permutation *p*	BH-FDR *q*	Partial Pearson *r*	Permutation *p*	BH-FDR *q*
Amaryllidaceae	*Serratia*	0.042	0.5982	0.8347	-0.070	0.2662	0.6713
Amaryllidaceae	*Sporosarcina*	-0.075	0.3595	0.6801	-0.041	0.5253	0.8168
Cucurbitaceae	*Arthrobacter*	-0.029	0.7303	0.8851	0.148	0.1126	0.4941
Cucurbitaceae	*Carnobacterium*	-0.098	0.2313	0.5223	-0.113	0.1325	0.4941
Cucurbitaceae	*Enterococcus*	-0.060	0.4681	0.7447	-0.041	0.6466	0.8704
Cucurbitaceae	*Lactobacillus*	-0.019	0.8255	0.9320	0.021	0.7817	0.9120
Cucurbitaceae	*Planomicrobium*	0.110	0.1807	0.4587	0.143	0.0502	0.4125
Cucurbitaceae	*Pseudomonas*	-0.026	0.7542	0.8851	0.094	0.2266	0.6254
Cucurbitaceae	*Psychrobacter*	0.019	0.8203	0.9320	-0.154	0.0738	0.4125
Cucurbitaceae	*Rahnella*	0.040	0.6201	0.8347	-0.041	0.5599	0.8168
Cucurbitaceae	*Serratia*	0.088	0.2750	0.5833	-0.008	0.9086	0.9785
Cucurbitaceae	*Sporosarcina*	0.003	0.9716	0.9716	0.009	0.8962	0.9785
Fabaceae	*Arthrobacter*	0.293	0.0004	0.0140	-0.008	0.9308	0.9872
Fabaceae	*Carnobacterium*	-0.067	0.4171	0.7105	0.004	0.9636	0.9931
Fabaceae	*Enterococcus*	0.201	0.0127	0.0924	-0.002	0.9790	0.9931
Fabaceae	*Lactobacillus*	0.128	0.1163	0.3700	-0.044	0.5989	0.8477
Fabaceae	*Planomicrobium*	-0.131	0.1060	0.3673	-0.046	0.6055	0.8477
Fabaceae	*Pseudomonas*	0.235	0.0051	0.0510	0.120	0.1969	0.5743
Fabaceae	*Psychrobacter*	-0.266	0.0010	0.0154	0.032	0.7047	0.8739
Fabaceae	*Rahnella*	-0.232	0.0046	0.0510	-0.137	0.0752	0.4125

Plant family	Bacterial genus	Unadjusted analysis			Sampling-group-adjusted analysis		
		Pearson *r*	Permutation *p*	BH-FDR *q*	Partial Pearson *r*	Permutation *p*	BH-FDR *q*
Fabaceae	*Serratia*	-0.188	0.0207	0.1207	-0.017	0.7980	0.9157
Fabaceae	*Sporosarcina*	-0.053	0.5141	0.7823	0.064	0.4039	0.7427
Brassicaceae	*Arthrobacter*	0.123	0.1357	0.3958	0.077	0.3412	0.7238
Brassicaceae	*Carnobacterium*	-0.035	0.6764	0.8633	-0.050	0.5523	0.8168
Brassicaceae	*Enterococcus*	-0.041	0.6144	0.8347	0.010	0.9066	0.9785
Brassicaceae	*Lactobacillus*	0.035	0.6686	0.8633	0.117	0.1341	0.4941
Brassicaceae	*Planomicrobium*	0.012	0.8757	0.9578	0.058	0.5086	0.8168
Brassicaceae	*Pseudomonas*	-0.068	0.4087	0.7105	-0.240	0.0133	0.4125
Brassicaceae	*Psychrobacter*	-0.109	0.1908	0.4606	-0.066	0.3926	0.7427
Brassicaceae	*Rahnella*	0.034	0.6783	0.8633	0.001	0.9931	0.9931
Brassicaceae	*Serratia*	0.078	0.3313	0.6442	0.091	0.1818	0.5533
Brassicaceae	*Sporosarcina*	0.061	0.4614	0.7447	0.060	0.4364	0.7563
Amaranthaceae	*Arthrobacter*	-0.009	0.9154	0.9617	0.004	0.9668	0.9931
Amaranthaceae	*Carnobacterium*	-0.132	0.1102	0.3673	-0.130	0.1229	0.4941
Amaranthaceae	*Enterococcus*	0.125	0.1277	0.3887	0.159	0.0586	0.4125
Amaranthaceae	*Lactobacillus*	0.085	0.3095	0.6372	0.050	0.5594	0.8168
Amaranthaceae	*Planomicrobium*	0.181	0.0267	0.1386	0.183	0.0276	0.4125
Amaranthaceae	*Pseudomonas*	-0.170	0.0365	0.1597	-0.136	0.1320	0.4941
Amaranthaceae	*Psychrobacter*	0.026	0.7587	0.8851	0.028	0.7453	0.8843
Amaranthaceae	*Rahnella*	-0.068	0.4117	0.7105	-0.037	0.6199	0.8508

Plant family	Bacterial genus	Unadjusted analysis			Sampling-group-adjusted analysis		
		Pearson *r*	Permutation *p*	BH-FDR *q*	Partial Pearson *r*	Permutation *p*	BH-FDR *q*
Amaranthaceae	*Serratia*	0.026	0.7495	0.8851	0.057	0.3913	0.7427
Amaranthaceae	*Sporosarcina*	-0.147	0.0751	0.2767	-0.175	0.0245	0.4125
Poaceae	*Arthrobacter*	0.016	0.8448	0.9387	0.148	0.0980	0.4900
Poaceae	*Carnobacterium*	0.005	0.9480	0.9617	-0.110	0.1522	0.5073
Poaceae	*Enterococcus*	-0.258	0.0011	0.0154	0.050	0.5601	0.8168
Poaceae	*Lactobacillus*	-0.199	0.0132	0.0924	-0.015	0.8413	0.9499
Poaceae	*Planomicrobium*	0.098	0.2258	0.5223	-0.086	0.2685	0.6713
Poaceae	*Pseudomonas*	0.066	0.4263	0.7105	0.093	0.3060	0.6910
Poaceae	*Psychrobacter*	-0.032	0.7115	0.8851	-0.145	0.0706	0.4125
Poaceae	*Rahnella*	0.165	0.0436	0.1795	0.080	0.2914	0.6867
Poaceae	*Serratia*	0.109	0.1835	0.4587	0.023	0.7182	0.8739
Poaceae	*Sporosarcina*	0.213	0.0079	0.0691	0.053	0.4430	0.7563

Note: All analyses included 150 matched fecal samples. Unadjusted associations were calculated using Pearson correlations of CLR-transformed compositions with 9,999 unrestricted permutations. Sampling-group-adjusted associations were calculated from residuals of taxon-specific linear models including sampling group as a categorical fixed effect, with 9,999 within-group permutations. Benjamini–Hochberg correction was applied across all 70 tests within each analysis; $q < 0.05$ was considered significant.
